# The Gag Cleavage Product, p12, is a Functional Constituent of the Murine Leukemia Virus Pre-Integration Complex

**DOI:** 10.1371/journal.ppat.1001183

**Published:** 2010-11-11

**Authors:** Adi Prizan-Ravid, Efrat Elis, Nihay Laham-Karam, Sara Selig, Marcelo Ehrlich, Eran Bacharach

**Affiliations:** 1 Department of Cell Research and Immunology, The George S. Wise Faculty of Life Sciences, Tel Aviv University, Tel Aviv, Israel; 2 Molecular Medicine Laboratory, Rambam Health Campus and Rappaport Faculty of Medicine, Technion, Haifa, Israel; University of Geneva, Switzerland

## Abstract

The p12 protein is a cleavage product of the Gag precursor of the murine leukemia virus (MLV). Specific mutations in p12 have been described that affect early stages of infection, rendering the virus replication-defective. Such mutants showed normal generation of genomic DNA but no formation of circular forms, which are markers of nuclear entry by the viral DNA. This suggested that p12 may function in early stages of infection but the precise mechanism of p12 action is not known. To address the function and follow the intracellular localization of the wt p12 protein, we generated tagged p12 proteins in the context of a replication-competent virus, which allowed for the detection of p12 at early stages of infection by immunofluorescence. p12 was found to be distributed to discrete puncta, indicative of macromolecular complexes. These complexes were localized to the cytoplasm early after infection, and thereafter accumulated adjacent to mitotic chromosomes. This chromosomal accumulation was impaired for p12 proteins with a mutation that rendered the virus integration-defective. Immunofluorescence demonstrated that intracellular p12 complexes co-localized with capsid, a known constituent of the MLV pre-integration complex (PIC), and immunofluorescence combined with fluorescent *in situ* hybridization (FISH) revealed co-localization of the p12 proteins with the incoming reverse transcribed viral DNA. Interactions of p12 with the capsid and with the viral DNA were also demonstrated by co-immunoprecipitation. These results imply that p12 proteins are components of the MLV PIC. Furthermore, a large excess of wt PICs did not rescue the defect in integration of PICs derived from mutant p12 particles, demonstrating that p12 exerts its function as part of this complex. Altogether, these results imply that p12 proteins are constituent of the MLV PIC and function in directing the PIC from the cytoplasm towards integration.

## Author Summary

All retroviruses reverse transcribe their RNA genome to a DNA copy in the cytoplasm of the infected cell. To be expressed, the viral genomic DNA has to travel to the cell nucleus and to integrate into the cellular chromosomes. This trafficking is governed by cellular and viral proteins that associate with the viral genome to form a ‘pre-integration complex’ (PIC), yet the full composition of this complex is unknown. Former studies showed that for the murine leukemia virus (MLV), mutations in a viral protein named p12 abrogate MLV infection, after reverse transcription and prior to the integration step, suggesting a role for this protein in early stages of infection. However, the precise mechanism of p12 action is not known. We combined microscopic, genetic and biochemical techniques to provide evidence that the p12 protein is part of the MLV PIC and that it exerts its function from within this complex. These analyses also suggest a role for p12 in the trafficking of the PIC from the cytoplasm to the chromosomes of the infected cell. Altogether, these findings highlight an important ‘building block’ of a complex that is essential for MLV infection.

## Introduction

Reverse transcription and integration are the hallmarks of the retroviral life cycle. These steps include reverse transcription of the genomic RNA into a linear double-stranded DNA and the subsequent integration of this DNA into the genome of the infected cell. These events are part of the ‘early’ stages of the retroviral life cycle, starting with the binding of the virus to its cellular receptor and ending once the integration step has occurred. Reverse transcription and integration are mediated by the viral enzymes; reverse transcriptase (RT) and integrase (IN), respectively; both are cleavage products of the polyprotein encoded by the viral *pol* gene. Reverse transcription occurs in a cytoplasmic complex, termed reverse transcription complex (RTC), which transforms to the PIC (reviewed in [1], [2]). The PIC harbors the viral DNA and travels from the cytoplasm to the nucleus, to target the chromatin of the infected cell for integration.

The full composition of the RTC and PIC is not known; this is true not only for the cellular components, but also for the viral constituents of these complexes [2], [3]. Some of the known cellular components identified in RTC/PIC of different retroviruses include: the barrier of auto-integration factor (BAF) [4], [5], high-mobility group proteins (HMGs) [6], [7], Ku [8], lamina-associated polypeptide 2α (LAP2α) [9], and lens epithelium-derived growth factor (LEDGF/p75) [10], [11]. To date, the viral protein components identified in the RTC/PIC of the simple MLV include: RT [12], nucleocapsid (NC) [13], capsid (CA) [12] and IN [7], [12], [13], [14]; while in the complex human immunodeficiency virus type-1 (HIV-1), NC [15], matrix (MA) [16], [17], [18], [19], RT [15], [16], [17], [18], [19], IN [6], [15], [16], [19], [20] and Vpr [15], [19] are present.

The trafficking of the PIC towards the chromatin of infected cells might be substantially variable between different retroviruses. This is demonstrated in part, by the fact that the nuclear entry by the MLV PIC is strictly dependent on mitosis [21], whereas nuclear entry by HIV-1 PIC can occur efficiently in nondividing cells [22], [23], [24], [25], [26]. Differences in PIC composition may contribute to such a variance; it has been shown that the MLV PIC contains the viral CA protein [14], while the HIV PIC does not [18]; this may contribute to the inability of wt MLV PICs to enter the nucleus through the nuclear pores [27], and render the MLV PICs dependent on nuclear envelope breakdown during mitosis for nuclear entry [21].

One viral protein thought to influence the trafficking of the MLV PIC, is the p12 protein. This protein is a cleavage product of the Gag precursor, which is the major structural protein in the virion. A few thousand Gag molecules assemble to form one viral particle, and these are cleaved by the viral protease during the virion maturation step. For MLV, this cleavage results in the release of the following viral proteins: MA, p12, CA and NC. The p12 domain is known to act in the late budding process of the Gag precursor, and accordingly mutations in the p12 hamper virion release [28], [29], [30], [31]. However, additional mutations in this protein have specifically affected early stages of MLV infections, revealing a critical role for p12 in these stages [3], [28], [31], [32], [33]. Analysis of a subset of these mutants revealed normal generation of the linear genomic DNA but no generation of circular viral DNA forms. Such circular forms are thought to be formed by nuclear enzymes from a portion of the linear viral DNA and although these circles are not substrates for the IN [34], [35], [36], they serve as a marker for nuclear entry by the viral DNA. Their absence during the infection of the p12 mutants suggested that p12 may function in directing the PIC into the nucleus or alternatively affects unknown nuclear steps needed for integration. Importantly, for at least one of these p12 mutants, the PIC appeared competent for integration in an *in vitro* assay [3]. A later study provided genetic evidence that p12 may function in concert with CA in early stages of infection: using swap mutants between the different domains of the Gag of MLV and its closely related virus, the spleen necrosis virus (SNV), it was demonstrated that productive infection can be achieved only when the p12 (or p18 from SNV) and CA domains are from the same virus [37]. Although these studies strongly indicate p12 participation in the early events of MLV infection, the precise mechanisms of p12 action is not known. Here we provide evidence that p12 is a part of the PIC and functions from within this complex in early stages of infection.

## Results

### Generation of replication-competent MLV with tagged p12 proteins

**Figure 1:**
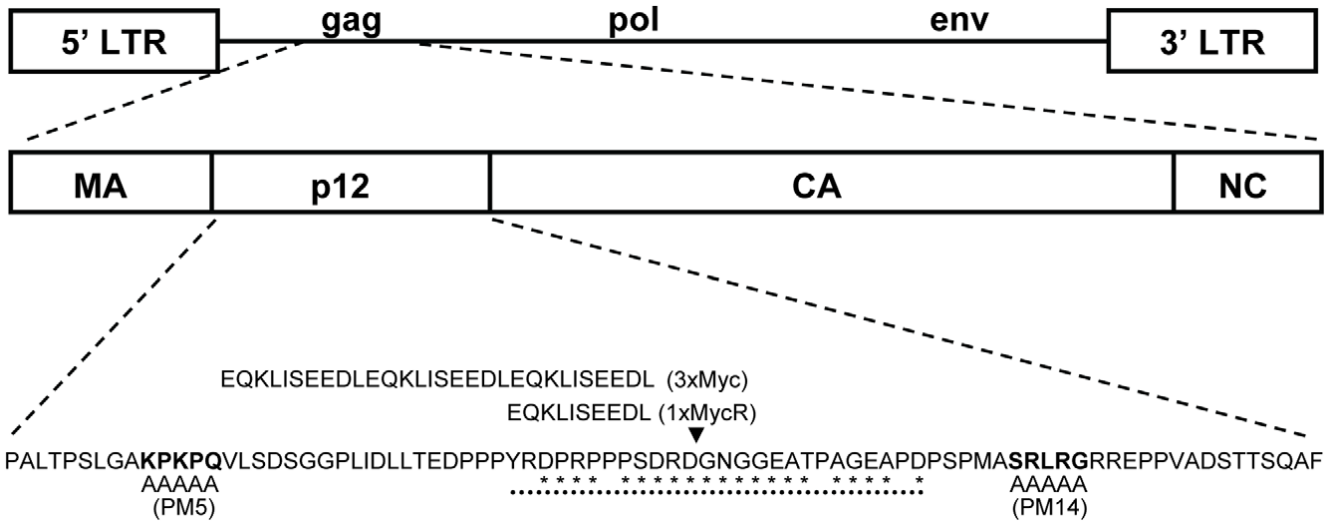
Schematic representation of MLV genome, p12 sequence and mutations. Schematic representation of MLV genome with an expanded view of the *gag* gene, demonstrating the MA, p12, CA and NC domains. The p12 protein sequence is aligned with the alanine blocks in PM5 and PM14 viruses, which replace the wt residues (in bold) in these mutants [31]. Insertion sites in p12 of the Myc epitope sequences are marked with an arrowhead. The black dots are aligned with a region that was found to tolerate mutations in previous studies [28], [31], and residues in this region that were found to be relatively variable by phylogenetic analysis (Materials and Methods) are marked with asterisks.

**Figure 2:**
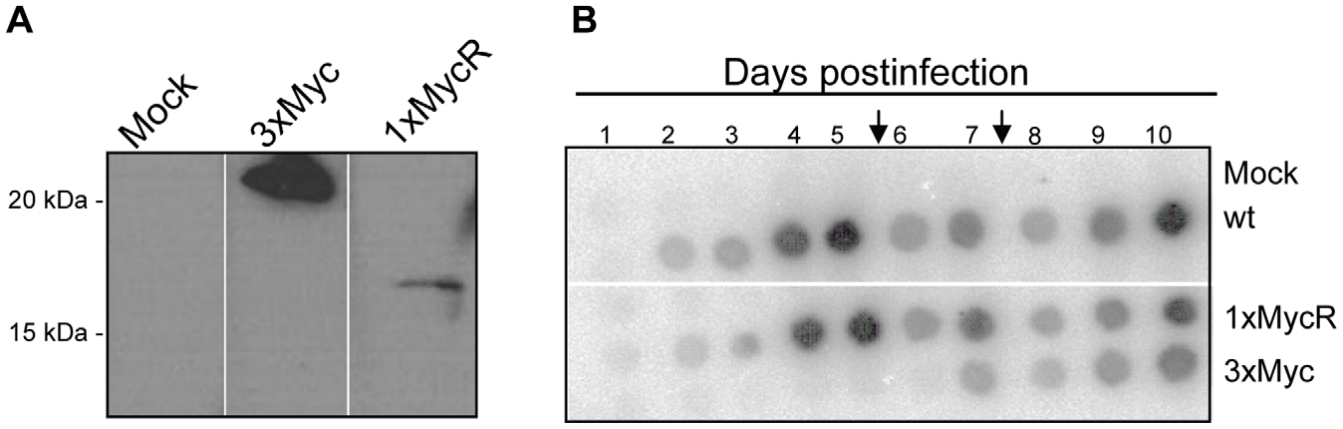
Characterization of virion production and replication of viruses with Myc-tagged p12 proteins. (A) Western blot analysis of Myc-tagged p12 proteins. Equal amounts of plasmids, coding for 3xMyc and 1xMycR viruses were transfected into 293T cells. Mock are cells transfected with no DNA. Virions were purified from culture supernatants two days posttransfection by ultracentrifugation through 25% sucrose cushions. Tagged-p12 proteins in virion pellets were analyzed by Western blot using anti-Myc monoclonal antibody (9E10). The migration of the cleaved Myc-tagged p12 protein is shown, highlighting the difference in size between the triple and single Myc-tagged p12. (B) Replication kinetics of wt and 1xMycR viruses. Naïve NIH3T3 cells were infected with equivalent amounts of wt or 1xMycR virions (normalized by RT activity) and replication kinetics were determined by radioactive exogenous RT assay. Culture supernatants were harvested at the indicated days postinfection and the arrows indicate the days at which the infected cultures were diluted. Virion level in the sample is directly correlated to the intensity of the radioactive signal [57]. Mock indicates infection with culture medium with no virus.

To study p12 function during the early stages of MLV life cycle, we constructed epitope-tagged p12 to enable its visualization in infected cells. Former mutagenesis studies have demonstrated that MLV tolerates mutations in the central region of p12 rather than in other parts of this protein [28], [31], [33], [38] (Fig. 1). In addition, our phylogenetic analysis revealed that the central region of p12 is relatively less conserved between members of the MLV family, further suggesting that this region may tolerate changes in its sequence (Fig. 1). We then inserted into this region a triple repeat of the Myc epitope (3xMyc; Fig. 1) to enhance detection of tagged p12. The p12 protein of this virus could easily be detected by Western blot analysis (Fig. 2A), but the replication of this mutant was greatly reduced in comparison to wt (Fig. 2B). However, we were able to recover a revertant virus with normal replication kinetics, after repeated passages of the 3xMyc virus on naïve NIH3T3 cells. A BsrGI-XhoI fragment of the revertant, containing the entire p12 domain and flanking Gag sequences, was PCR amplified and sequenced, revealing the existence of only one in-frame copy of the Myc epitope in p12 with no other mutations. This fragment was used to replace the wt sequence between the BsrGI-XhoI restriction sites in a molecular clone of MLV (pNCS, Materials and Methods), and the resulting virus was named 1xMycR (Fig. 1). Further analysis of purified virions of the 1xMycR virus demonstrated that its p12 could be detected with anti-Myc antibodies by Western blot analysis and, as expected, the protein had a reduced molecular weight compared to p12 of the 3xMyc virus (Fig. 2A). Importantly, unlike the 3xMyc virus, the 1xMycR virus replicated with wt-like kinetics in NIH3T3 cells (Fig. 2B), demonstrating that the reduction in the number of repeats of the epitope tag in p12, accounted for the improved replication of the latter virus.

### Detection of the 1xMycR p12 in early stages of MLV infection using immunofluorescence

**Figure 3:**
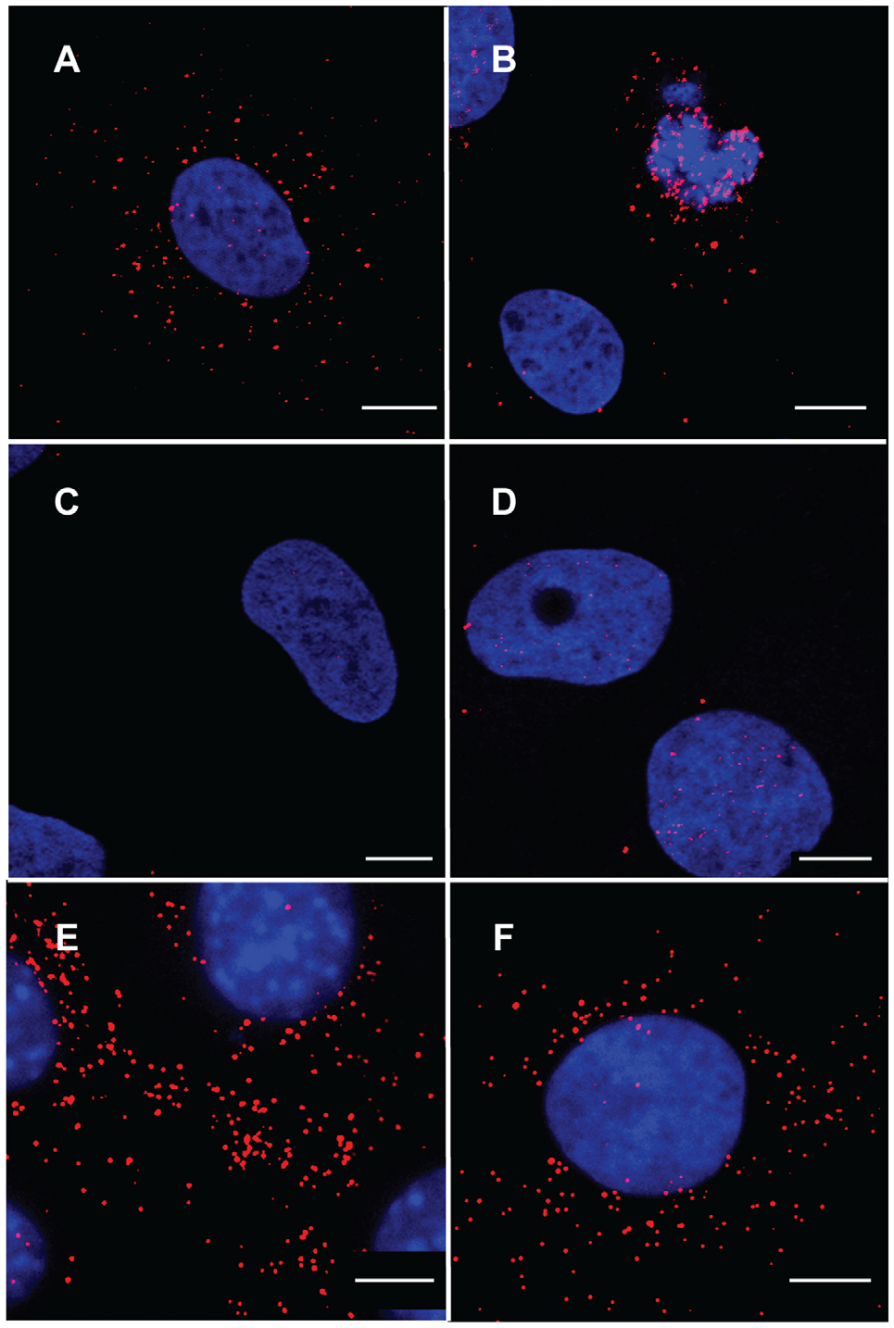
Immunofluorescence analysis of 1xMycR or wt -infected cells. Representative images of U/R (A, B, C and F), U2OS (D), or NIH3T3 (E) cells that were infected with 1xMycR (A, B, D, E and F) or wt (C) viruses. 12 h postinfection the cells were stained with DAPI, anti-Myc monoclonal antibody and a secondary, Cy-3-conjugated, anti-mouse antibody. Serum starvation and aphidicolin treatment were applied to arrest the cell cycle prior and during the infection (F). Cultures were visualized with LSM 510 META confocal microscope (Zeiss) (A, B, C, D and F), or with spinning disk confocal (Yokogawa CSU-22 Confocal Head) microscope (Axiovert 200 M, Carl Zeiss MicroImaging) (E). Bars represent 10 µm.

Next, we examined whether p12 proteins can be detected by immunofluorescence in 1xMycR-infected cells, using anti-Myc antibodies. To unambiguously distinguish signals resulting from authentic infection and background signals, we preformed the infections with human cells (osteosarcoma; U2OS) that cannot be normally infected due to the lack of the MLV receptor; or with a cognate, U2OS-derived, cell line (U/R) that stably expresses the mCAT-1 murine receptor for MLV [39], and is susceptible to MLV infection (Materials and Methods). Parental U2OS or U/R cells were infected either with 1xMycR or wt viruses, fixed and immunostained with anti-Myc antibody. Clear punctate fluorescence could be detected in the cytoplasm of 1xMycR infected U/R cells (Fig. 3A). We also observed a punctate staining of p12 that was in close proximity to the chromosomes, in dividing cells that were identified by their condensed chromosomes (Fig. 3B and see below). In the control U/R cells, p12 staining was not observed following infection with the wt virus (Fig. 3C); and very low p12 staining was observed in 1xMycR-infected parental U2OS cells (Fig. 3D), likely representing a low level of adherence of the virus to the cells. In addition, no immunofluorescence was observed in mock-infected U/R cells or in 1xMycR-infected U/R cells that were stained with the secondary, but not with the primary anti-Myc antibody (data not shown). Thus, the majority of the punctate staining represents a signal that is derived from an authentic infection, and not from background staining or non-specific adherence of viral particles or antibodies to the cell membrane. Further support to this idea came from the direct correlation that was found between the number of p12 puncta in infected cells and the amount of 1xMycR virions that were used for infection (Fig. S1), suggesting that these dots represent p12 proteins that originated from physical particles. The punctate pattern of staining as opposed to a diffuse staining, suggests that the p12 molecules are associated in a complex. Similar punctate staining was observed when cells derived from the natural host of MLV (murine NIH3T3 cells) were infected with the 1xMycR virus (Fig. 3E). Of note, whereas clear immunofluorescence signal was detected in 1xMycR-infected cells, no such signal was observed in cells that were infected with the previously described replication-competent MLV that contained a Flag-tagged p12 [33], and that were stained with anti-Flag antibodies (data not shown). This discrepancy may result from the different locations of the tags in p12 and from the fact that the Flag epitope replaced few residues in p12, while the Myc epitope was inserted into the complete sequence of this protein, rendering this tag in the in 1xMycR virus more accessible to the antibodies.

### Detection of p12 in early stages of infection in dividing and cell-cycle arrested cells

The early detection of p12 at 12 h postinfection (Fig. 3), suggested that the immunofluorescence detected the p12 proteins of the incoming virus and not the p12 domain of Gag precursors that were synthesized in late stages of the infection. To further examine this issue, we arrested the cell cycle of U/R cells by serum starvation and aphidicolin treatment, as this procedure was shown to block nuclear entry and integration of the incoming MLV, averting Gag synthesis in later stages of infection [21]. We then infected the cells with 1xMycR virus and observed the same punctate fluorescent signal for p12 in the aphidicolin-treated cells (Fig. 3F), confirming that p12 staining is of the incoming virus and not of newly synthesized Gag precursors.

**Figure 4:**
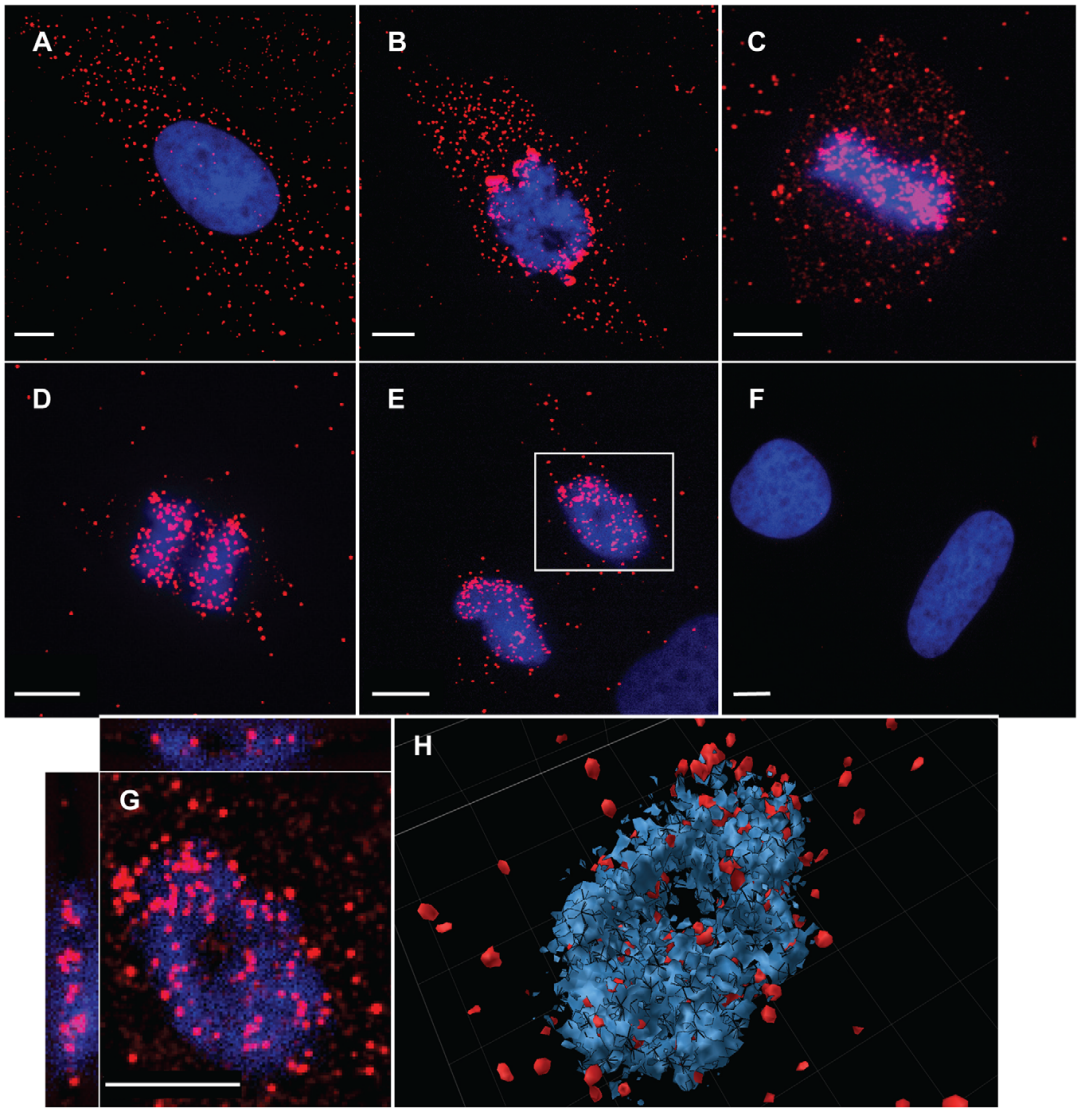
Immunofluorescence analysis of p12 proteins at different stages of the cell cycle. Dividing, non-synchronized U/R cells were infected with 1xMycR (A–E and G–H) or with wt (F) viruses. 12 h postinfection the cells were stained as described in Fig. 3 and visualized with spinning disk confocal (Yokogawa CSU-22 Confocal Head) microscope (Axiovert 200 M, Carl Zeiss MicroImaging). Stages of the cell cycle were determined according to the condensation and separation of the chromosomes. A and F, interphase; B–E, prometaphase, metaphase, early and late anaphase, respectively. G and H; single and serial optical sections of the chromosomes that appear in the inset in E, respectively. The serial sections were reconstituted into a 3D image. Bars represent 10 µm.

The p12 staining in aphidicolin-treated cells (Fig. 3F) was mainly restricted to the cytoplasm, as was observed for cells that were not treated with aphidicolin and that were in the interphase stage of the cell cycle (Fig. 3A). In contrast, p12 was detected in both the cytoplasm and in the vicinity of chromosomes of dividing cells (Fig. 3B). To further study and quantify this observation, cycling non-synchronized U/R cells were infected with 1xMycR virus and 12 h postinfection the cells were fixed and stained for p12 and the cellular DNA as described above. Mitotic cells were identified on the basis of chromosome condensation and the typical configuration of each mitotic stage. In addition, the extent of p12 staining (red) that overlapped the staining of cellular DNA (blue) was determined (see Materials and Methods), to quantify the distribution of p12 proteins between the cytoplasm and the chromosomes. Fig. 4 shows images of 1xMycR-infected cells at different stages of the cell cycle, and the quantification of p12 distribution is represented in Fig. S2. As can be seen, p12 was mainly detected in the cytoplasm of interphasic cells (Fig. 4A) with only 6% overlap between p12 and the chromosomes (Fig. S2), similar to what was shown above (Fig. 3A and F). In contrast, cells at different stages of mitosis showed a much higher overlap between the p12 and the chromosomes (Fig. 4B–E); the percentages of this overlap were quantified to be 49, 75, 68 and 72% at prometaphase, metaphase, as well as early and late anaphase, respectively (Fig. S2). No p12 signal was detected in U/R cells that were infected with wt virus and used to control for the immunofluorescence specificity (Fig. 4F). Overall, complexes of p12 proteins appeared to migrate from the cytoplasm to the nucleus and to accumulate at the vicinity of the chromosomes in dividing cells.

To further study the association of p12 with the chromosomes, optical sections were generated for the mitotic chromosomes in infected cells (inset, Fig. 4E). This microscopy analysis clearly demonstrated that p12 proteins were detected at the same plane of the chromosomes (Fig. 4G). Reconstitution of the serial optical sections into a 3D image further showed the close proximity between p12 proteins and the chromosomes (Fig. 4H). Thus, p12 proteins appear to be closely associated with, and even imbedded in, the mitotic chromosomes of infected cells.

### p12 co-localize with CA, but not with MA, in infected cells

**Figure 5:**
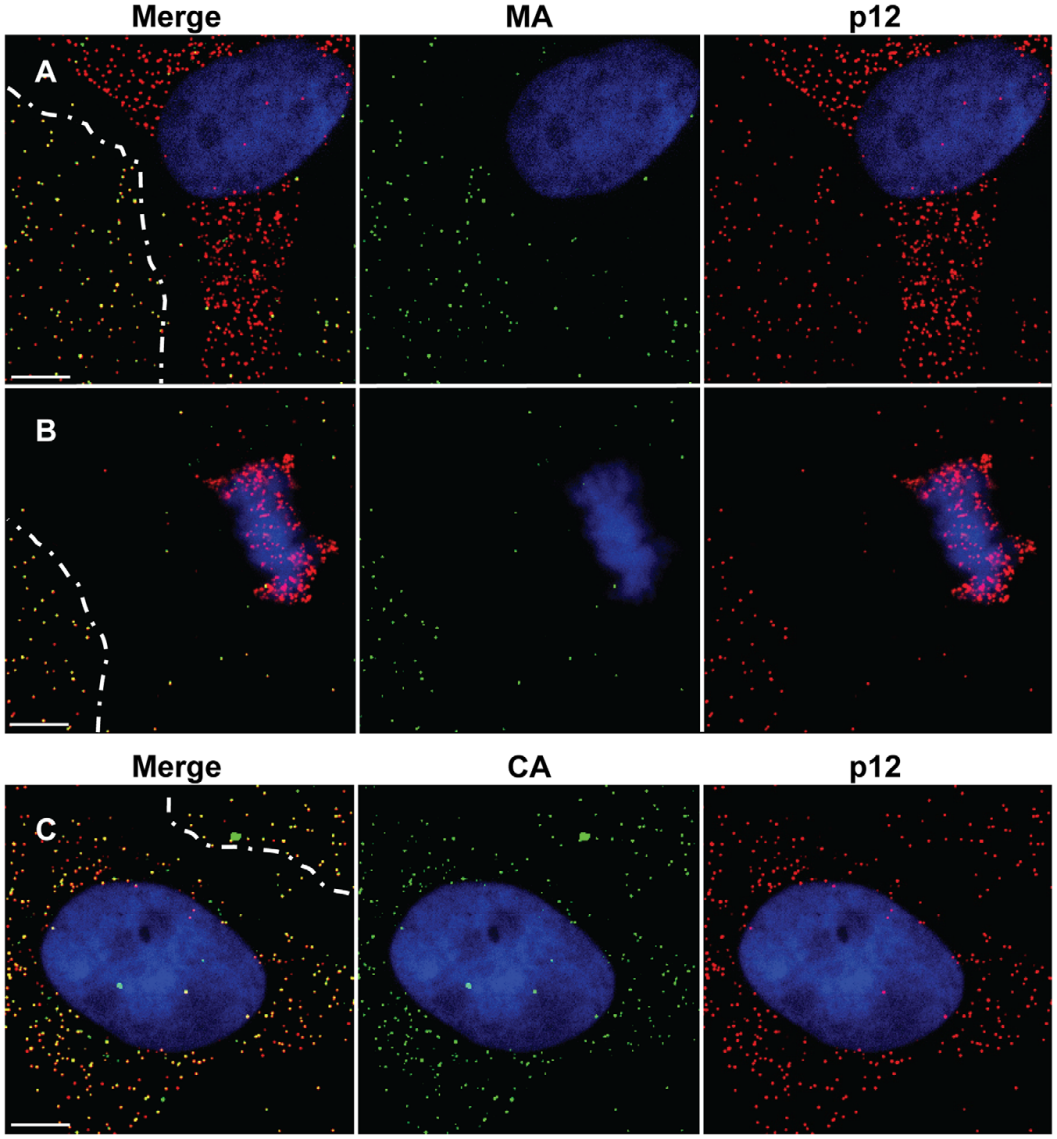
Analysis of the co-localization of p12 with MA or CA. U/R cells were infected with the 1xMycR virus and analyzed by immunofluorescence, as described in Fig. 3. p12 (A–C), MA(A, B) and CA (C) were detected with the anti-Myc monoclonal antibody, goat anti-MA and goat anti-CA polyclonal antibodies, respectively, and the matched secondary FITC-conjugated donkey anti-mouse IgG and Red-X- conjugated donkey anti-goat IgG. For consistency, the images were pseudocolored to present p12 in red and MA or CA in green. Dashed lines mark the boundaries of cell-free regions where virions were detected attached to the cover glass. Bars represent 10 µm.

The above results implied that p12 proteins are found in complexes that migrate from the cytoplasm to the chromosomes in dividing cells. These resemble the characteristics of the PIC [21], suggesting that p12 may be a constituent of this complex. If this notion is true then in infected cells p12 should co-localize with components of the PIC but not with virion constituents that are not part of the PIC. To test this, we performed immunofluorescence analysis to detect the spatial relationships between p12 and MA - a virion constituent that is thought to become dispersed in the infected cell upon the uncoating step and is not known to be part of the MLV PIC; and CA - a virion constituent that is part of the MLV PIC [12]. 1xMycR-infected U/R cells were stained with anti-Myc antibodies, together with anti- MA or CA antibodies. This analysis revealed that, as expected for virion constituents, the p12 (red fluorescence) and MA (green fluorescence) proteins co-localized (yellow fluorescence) in particles that adhered to the cover glass in regions that were free of cells (Fig. 5A, B). In contrast, no such overlap could be detected between the p12 and MA in the infected cells. The lack of co-localization between p12 and MA in the infected cells was further emphasized in conditions where p12 proteins migrated towards the mitotic chromosomes (Fig. 5B). When the distribution of CA and p12 was tested, co-localization of the two proteins was clearly detected both in virions that adhered to the glass outside the cell and in the infected cell (Fig. 5C). Altogether, these immunofluorescence analyses demonstrated that in the virions p12 proteins are co-localized with MA and CA, yet in the infected cells p12 is associated with the CA but not with the MA proteins, suggesting that p12 puncta in infected cells are derived from uncoated virions and not from internalized virus particles, and further hinting for p12 association with the PIC.

### p12 and the viral DNA co-localize in infected cells

To further address the possibility that p12 molecules are indeed part of the MLV PIC we set to visualize the PICs in infected cells and to test the possible co-localization of p12 with these PICs. Since the presence of the viral DNA genome is the hallmark of the PIC, its detection by FISH has been used to identify MLV PICs by microscopy [21]. Thus, we aimed to detect the incoming PICs in early stages of infection, using DNA FISH in combination with immunofluorescence to define the time-dependent spatial correlation between the viral genomic DNA and the p12 protein.

**Figure 6:**
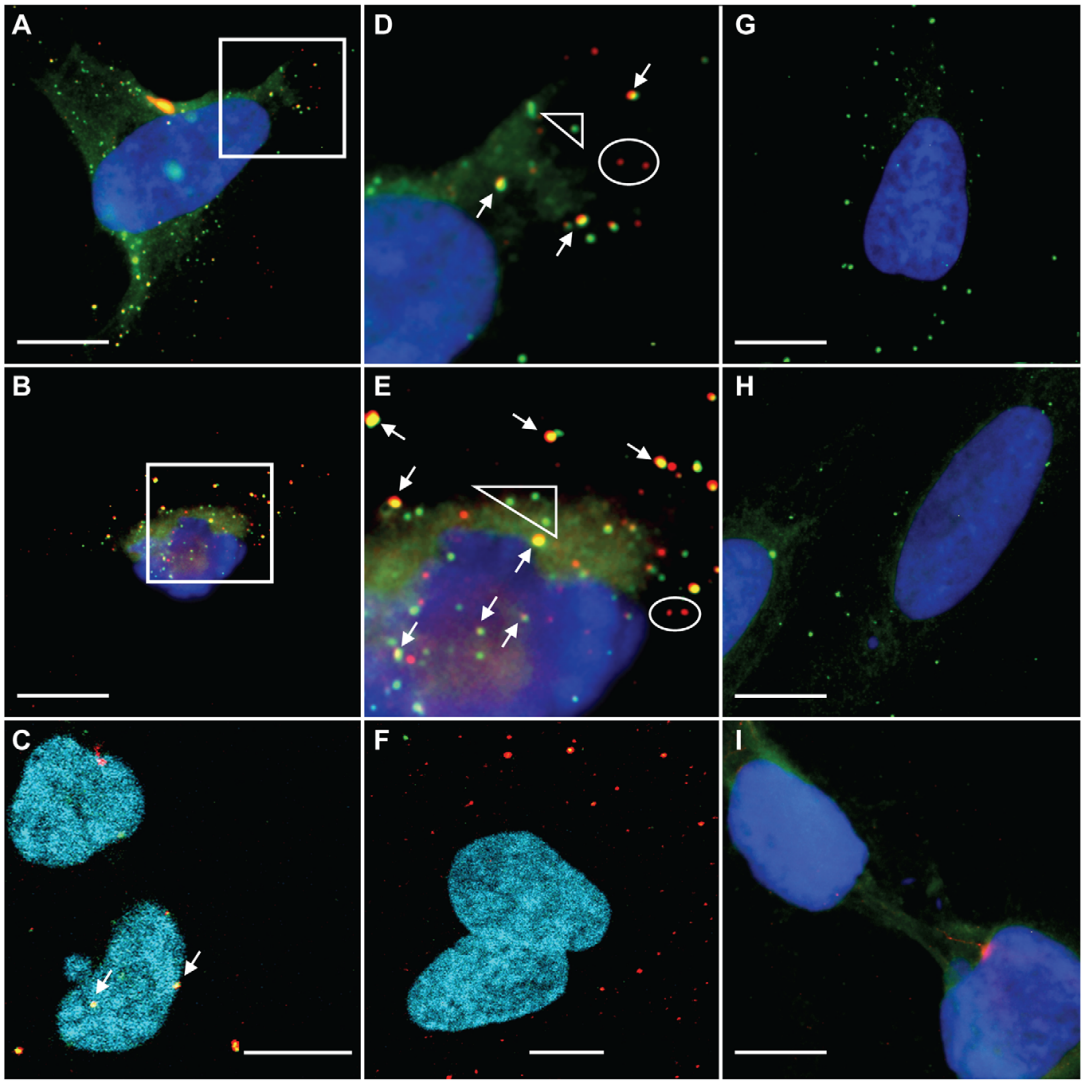
Detection of viral p12 proteins and genomic DNA by immunofluorescence combined with FISH. U/R cells (A–H) or U20S (I) were infected with 1xMycR (A–G, I) or wt (H) viruses, and 12 h (A–E, G–I) or 2 h (F) postinfection the cells were processed for immunofluorescence and FISH analysis. Myc-labeled p12 proteins were detected with a primary anti-Myc monoclonal antibody (A–F, H–I) and a secondary Cy-3-conjugated anti-mouse antibody (A–I). The immunodetection in G was performed with only the secondary, but not the primary, antibodies. The genomic DNA was detected with MLV-derived biotin-labeled probes and a FITC-conjugated avidin (A–I). D and E are magnifications of the insets in A and B, respectively. Arrows indicate overlaps between the viral p12 proteins and the genomic DNA signals. Ellipses and triangles show non-overlapping signals of the p12 and the genomic DNA, respectively. Images A, B, D, E and G–I were taken with a BX50 microscope (Olympus) or with a LSM 510 META confocal microscope (Zeiss) (C and F). Bars represent 10 µm.

For this analysis we used U/R cells, to avoid the problem of cross-hybridization with endogenous MLV-like elements found in mouse cells [40]. In addition, to avoid detection of carry-over of MLV plasmid DNA from transfected cells, we used viruses from chronically infected NIH3T3 cells. In preliminary FISH experiments we could readily detect clear and punctate fluorescence staining in U/R, but not in U2OS, cells that were infected with wt virus and that were hybridized with a MLV-derived, biotin-labeled probe that was detected with a Cy3-conjugated avidin, demonstrating the specificity of the detection method (Fig. S3 and see below). This staining was also reminiscent of the previously reported staining of MLV PICs by FISH in Rat-1 cells [21]. We then established conditions for immunofluorescence combined with DNA FISH (see Materials and Methods). U/R or U2OS cells were infected with 1xMycR or wt viruses, and 12 h postinfection, cells were stained with anti-Myc antibody and a secondary Cy3-conjugated antibody. FISH analysis followed, using a MLV-derived biotinylated probe and FITC-labeled avidin for the detection of the probe. A clear overlap (yellow) between the p12 proteins (red) and the MLV genomic DNA (green) was observed in U/R cells infected with 1xMycR virus (Fig. 6A–E). This overlap could be observed in the cytoplasm of infected cells (Fig. 6A, B, D and E), as well as in the vicinity of chromosomes (Fig. 6C), including condensed chromosomes of mitotic cells (Fig. 6B, E and S4). In contrast, such broad overlapping signals were absent in 1xMycR-infected U/R cells that were processed for immunofluorescence/FISH analysis at two hours postinfection (Fig. 6F). In this setting, only extensive punctate staining of p12 was observed, probably reflecting the lack of complete reverse transcription at this early time point. Quantification of more than 600 fluorescent dots in infected cells (Fig. S5) confirmed the above observations: at 12 h postinfection approximately 60% of the fluorescent puncta showed an overlapping signal between the p12 proteins and the viral genomic DNA, while approximately only 10 and 30 percents of the fluorescent dots showed such an overlap at earlier time points (2 and 6 h postinfection, respectively). In addition, at 12 h post infection about 10% of the total fluorescent dots, showed overlapping p12 and genomic DNA signals that could be located with the chromosomes. In contrast to these results, more than 99% of the extracellular dots (representing extracellular virions attached to the glass in cell-free regions, see Fig. 5), showed only p12 staining both at early (2 h) and late (12 h) time point postinfection (Fig. S5), likely due to the absence of efficient reverse transcription in these particles. Additional negative controls showed no overlapping signals between the p12 and the genomic DNA staining further emphasizing the genuineness of this analysis. These controls included: 1xMycR-infected U/R cells that were processed as above but without the addition of the primary anti-Myc antibody, showing only the green FISH signal (Fig. 6G); U/R cells, infected with the wt virus that lacks the Myc epitope, showing only the green FISH signal (Fig. 6H) and 1xMycR-infected U2OS cells, showing neither red nor green punctate fluorescence (Fig. 6I). Thus, the overlapping staining in 1xMycR-infected U/R cells, which was absent from the extracellular particles and from the negative controls, suggested that p12 proteins associate with viral genomic DNA and hence, p12 is indeed a component of the MLV PIC. Moreover, the overlap between p12 protein and viral DNA that was detected in close proximity to the condensed chromosomes (Fig. 6B, E and S5) suggested that p12 proteins escort the viral DNA until very close to the chromatin of dividing cells.

As was suggested above, the absence of intracellular overlapping immunofluorescence/FISH signals at two hours postinfection (Fig. 6F) likely reflected the lack of complete reverse transcription at this early time point, and may further suggest that reverse-transcription is not a prerequisite for the formation of p12 puncta in infected cells. We further investigated this point by the generation of MLV virus-like particles (VLPs), with or without the genomic RNA, and that their p12 proteins were Myc-tagged as the 1xMycR virus (Materials and Methods). These VLPs were then used to infect U/R cells, which were examined by immunofluorescence for the generation of p12 puncta. This analysis revealed a similar formation of p12 puncta, and similar accumulation of these dots close to mitotic chromosomes, for both conditions (Fig. S6). These results suggest that the presence of the genomic RNA, and its subsequent reverse transcription, are not a prerequisite for the formation and migration of p12 puncta in infected cells.

### p12 proteins function as part of the PIC

It should be noted that in 1xMycR-infected U/R cells, we also observed signals for the viral DNA and the p12 proteins that did not overlap (Fig. 6D and E; triangles and ellipses, and Fig. S5). Since only a fraction of the incoming viruses establish a productive infection [2], [41], [42], our microscopic analysis cannot resolve between the following two options: 1) only PICs that include functional p12 proteins have the potential to complete the early steps of infection or; 2) PICs that do not contain p12 are the infectious ones. Since p12 is crucial for MLV infection [3], [31], [33], in the latter scenario, p12 proteins function not as part of the PIC but autonomously, outside of this complex.

**Figure 7:**
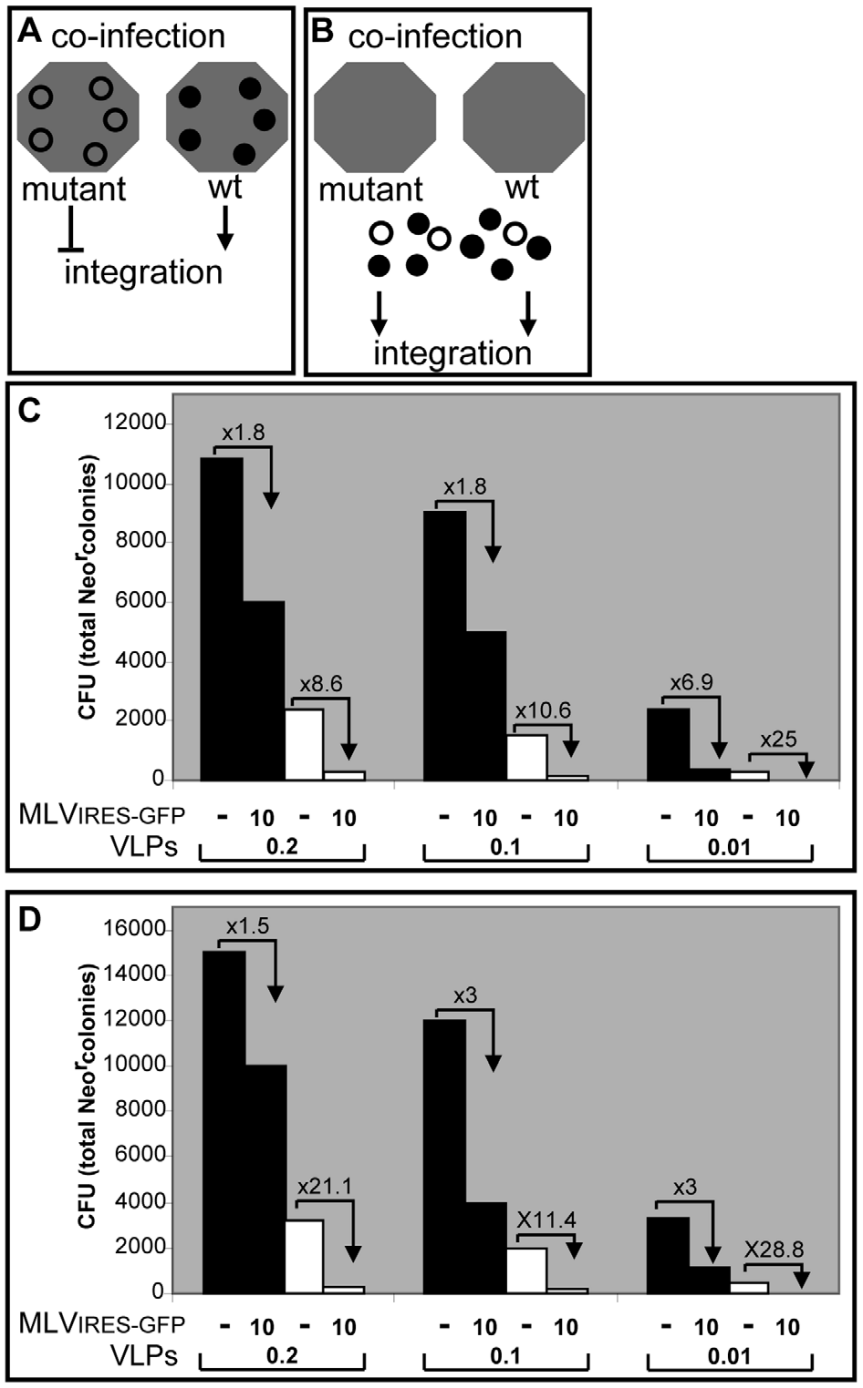
A complementation assay between wt and mutant p12 proteins. Schematic representation of the expected outcome of co-infection experiments with wt and p12 mutant viruses if p12 functions as a constituent of the PIC (A), or autonomously of the PIC (B). Gray hexagons represent PICs and black or empty circles represent wt or mutant p12 proteins, respectively. Arrowhead or T-shaped arrow represents productive or abortive integration, respectively. The total number of Neo^r^ colonies (colony forming units; CFU) obtained from NIH3T3 cells that were infected with the wt (black bars) or the PM14 mutant (white bars) VLPs carrying the Neo^r^ vector, and pseudotyped with the ecotropic MLV envelope (C) or the VSV-G glycoprotein (D) are shown. The combination of the MOIs of the MLV_IRES-GFP_ virus and the VLPs are indicated below the bars. The numbers over the broken arrows indicate the fold reduction in the CFU of the VLPs due to the co-infection with the MLV_IRES-GFP_ virus.

To address the question whether p12 modifies PIC function as part of the PIC or by acting separately from the PIC, we designed and applied a genetic ‘rescue’ assay based on complementation of wt and mutant p12 proteins. If p12 functions as a constituent of the PIC, upon co-infection of wt and p12 mutant viruses the wt p12 proteins should not rescue the defect in integration of PICs derived from the p12 mutant virus (Fig. 7A). In contrast, rescue should occur if p12 proteins act autonomously of the PIC, particularly in conditions where wt p12 proteins are present in large excess over mutant p12 proteins (Fig. 7B). Two types of MLV particles were generated for this assay: The first, named MLV_IRES-GFP_, was expressed from the pNCA_IRES-GFP_ clone, a replication-competent MLV with a GFP marker [43], allowing the accurate determination of its titer. MLV_IRES-GFP_ expresses wt p12 proteins. The second type was made of VLPs that were generated from helper plasmids expressing the MLV Gag with or without the PM14 mutation in p12 (Fig. 1), and the Pol and Env (ecotropic) proteins; these VLPs also encapsidated the pQCXIN retroviral vector (Clontech), expressing the neomycin resistance gene (Neo^r^). Importantly, pQCXIN is a self-inactivating vector due to a deletion in the U3 sequence of the 3′ LTR, which renders the vector compatible for only a single cycle of infection even in the presence of replicating virus such as the MLV_IRES-GFP_. We chose the PM14 mutation for this analysis since a virus that carries this mutation is capable of normal reverse transcription but is defective in integration [31]; yet, in our hands, VLPs with the PM14 mutation could transduce the pQCXIN vector at low but detectable efficiencies, allowing the quantification of p12 function in this complementation assay.

NIH3T3 cells were infected with dilutions of wt VLPs (MOI of 0.2, 0.1 or 0.01), or PM14 VLPs (with RT activities that matched the ones of the wt VLPs dilutions), in the presence or absence of MLV_IRES-GFP_ (MOI of 10). In these settings, the wt p12 proteins, derived from MLV_IRES-GFP_, are present in large excess over decreasing amounts of VLP-derived p12 proteins (wt or mutant) in infected cells. Infected cultures were then selected in G418- containing medium for two weeks, after which the number of Neo^r^ colonies was determined (Fig. 7C). The results of this experiment showed that in all tested ratios, no increase in the number of Neo^r^ colonies was observed for the PM14 VLPs (Fig. 7C, white columns) in the presence of MLV_IRES-GFP_, indicating that p12 proteins act from within the PIC. In fact, co-infection of MLV_IRES-GFP_ with either wt (Fig. 7C, black columns) or mutant VLPs resulted in a reduction in the number of Neo^r^ colonies; the level of this decrease augmented with the increase of MLV_IRES-GFP_/VLP ratio. This phenomenon likely represents competition between MLV_IRES-GFP_ and the VLPs over cellular factor(s) needed to establish the infection. Of note, the reduction in the number of Neo^r^ colonies in the presence of MLV_IRES-GFP_ was greater for the PM14 VLPs, compared to the wt VLPs, at all the tested ratios. For example, when the presence of MLV_IRES-GFP_ reduced the infectivity of wt VLPs by less than two-fold, a reduction of more than eight-fold was observed for PM14 VLPs. These results suggest that the PM14 mutant is more sensitive to the competition exerted by the MLV_IRES-GFP_ virus.

Similar results were obtained when we repeated this experiment using VLPs (wt and PM14) that were pseudotyped with the vesicular stomatitis virus G glycoprotein (VSV-G) instead of the ecotropic MLV envelope protein, to avoid direct competition between the VLPs and the MLV_IRES-GFP_ virus on the mCAT-1receptor, (Fig. 7D). No rescue of the infectivity of the p12 mutants was observed when these experiments were repeated using different conditions that included lower MOIs, the use of wt VLPs (instead of the replicating MLV_IRES-GFP_ virus) as a source for the wt p12 proteins, and PM14 VLPs encapsidating a vector with a different selection marker (data not shown). Overall, these experiments provide genetic evidence that p12 functions as part of the PIC.

### Co-immunoprecipitation (Co-IP) of p12 proteins and the viral genomic DNA and CA proteins

**Figure 8:**
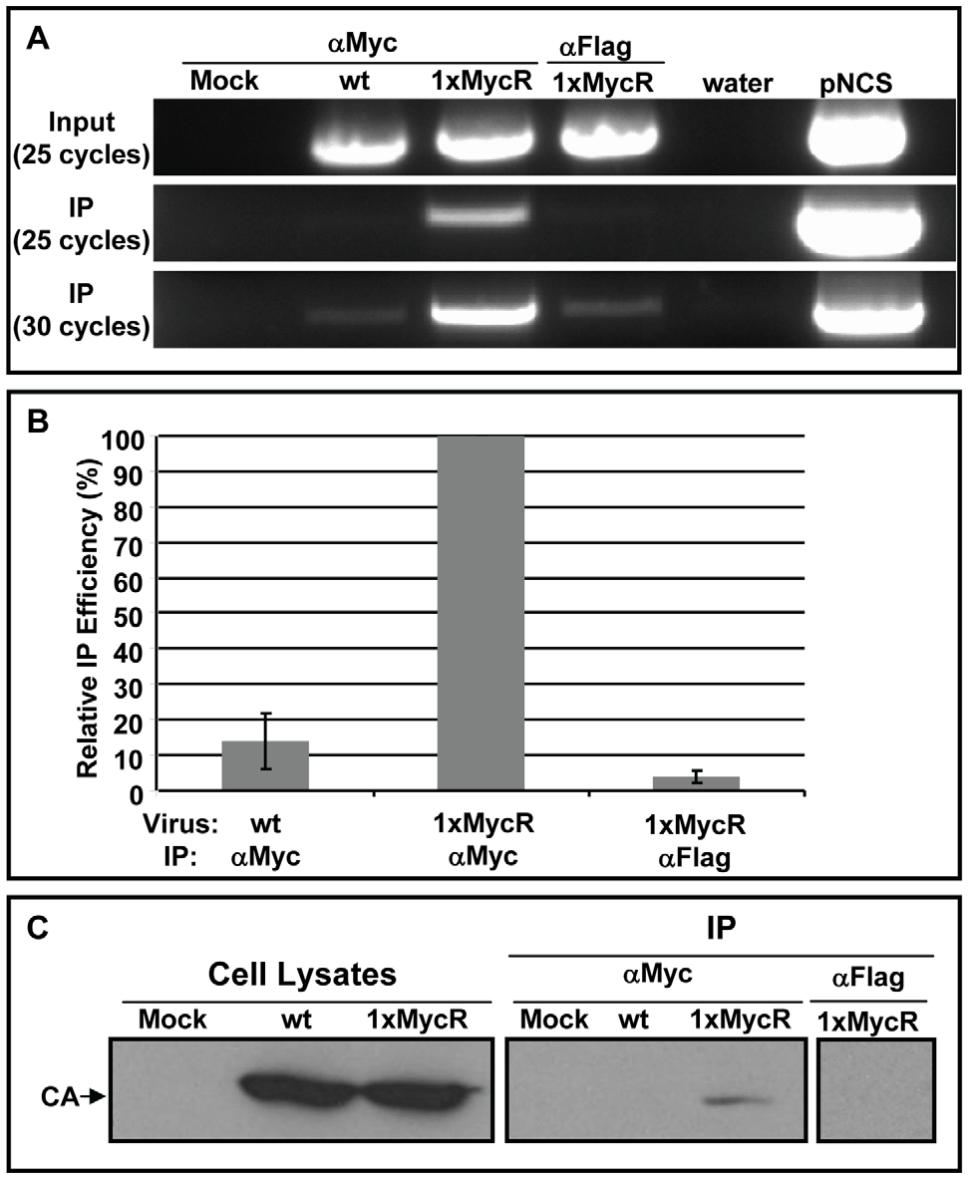
Co-IP of p12 with the viral genomic DNA and CA proteins. Lysates were prepared from NIH3T3 cells, infected with wt or 1xMycR viruses. To detect the viral genomic DNA (A and B) the lysates were incubated with anti-Myc (labeled ‘αMyc’), or control anti-Flag antibodies (labeled ‘αFlag’); both conjugated to protein G magnetic beads. Viral genomic DNA was PCR amplified from cell lysates (labeled ‘Input’) and from the magnetic beads (labeled ‘IP’) with MLV specific primers. After 25 and 30 cycles of amplification, the PCR products (875 bp long) were electrophoresed in 1% agarose gels containing ethidium bromide (A). ‘Mock’ indicates mock-infected NIH3T3 cells. Water and plasmid encoding the MLV genome (‘pNCS’) were used as negative and positive PCR controls, respectively. (B) The ‘Relative IP efficiency’ of the viral genomic DNA was quantified by qPCR (Materials and Methods) and is presented as the means ± the standard error of the means, obtained from three independent experiments. The qPCR values are presented in Fig. S7A. To test for CA immunoprecipitation (C), NIH3T3 were infected as in (A) and 15% of the indicated cell lysate was used to determine total CA levels (labeled ‘Cell Lysates’). The remaining lysates (labeled as in A) were used for IP with anti-Myc, or control anti-Flag monoclonal antibodies, bound to agarose beads. Cells exposed to medium with no virus served as a mock control. Pellets (labeled ‘IP’) and cell lysates were analyzed by Western Blot, using anti-CA polyclonal antibodies.

As mentioned above, the exact composition of retroviral PIC is not known, however the presence of the viral genomic DNA is the hallmark of the PIC. To further demonstrate that p12 is a component of the PIC we aimed to co-immunoprecipitate the viral genomic DNA with the p12 proteins. To immunoprecipitate p12, we infected NIH3T3 cells with wt or 1xMycR virus, lysed the cells and performed IP with anti-Myc, or control anti-Flag monoclonal antibodies. We then analyzed the cell lysates and the immunoprecipitates for the presence of the viral genomic DNA by PCR. This analysis revealed preferential IP of the genome of the 1xMycR virus when anti-Myc antibodies were used, compared to the controls (Fig. 8A). To better quantify this, we measured the amount of the viral DNA genome in the cell lysates and the immunopellets by quantitative PCR (qPCR) and calculated the relative efficiency of this IP (Fig. 8B and S7A). The average IP efficiency, obtained from three independent experiments, showed that when anti-Myc antibodies were used, the genome of the 1xMycR virus was immunoprecipitated approximately 7 fold higher than the genome of the wt virus. In addition, the average IP efficiency of the 1xMycR genome by the anti-Myc antibodies was approximately 25 fold higher than the one obtained for IP using anti-Flag antibodies. This specific IP of the viral genomic DNA with antibodies against p12 strongly suggested that p12 is indeed part of the PIC.

Since CA was identified as a component of the MLV PIC [14] we also tested if this protein co-immunoprecipitates with the p12 proteins. Co-IP experiments were performed similarly to what was described above for the IP of the viral genomic DNA. The presence of CA in cell lysates and in immunopellets was determined by Western blot analysis with polyclonal antibodies against CA (Fig. 8C). This analysis revealed that CA could be detected in the precipitate only when the precipitation included the lysates of the 1xMycR-infected cells and the anti-Myc antibodies; CA protein was absent from control precipitates obtained from wt-infected cell lysates that were reacted with anti-Myc antibodies, or from lysates of 1xMycR-infected cells that were reacted with the control anti-Flag antibodies.

To verify that the detected p12-CA interaction reflects an authentic, intracellular interaction and not an interaction present in internalized virus particles, we carried out an analogous pull-down experiment on both extracellular virions and infected cells. The results of this experiment clearly demonstrate that CA of the 1xMycR virus was immunoprecipitated by anti-Myc antibodies only from lysates of infected cells and not from lysates of extracellular virions (Fig. S7B and C). These results indicate co-association of CA - a known component of the MLV PIC - and p12 proteins, further providing evidence that p12 is indeed a component of the PIC.

### Mutant p12 proteins fail to traffic towards mitotic chromosomes

**Figure 9:**
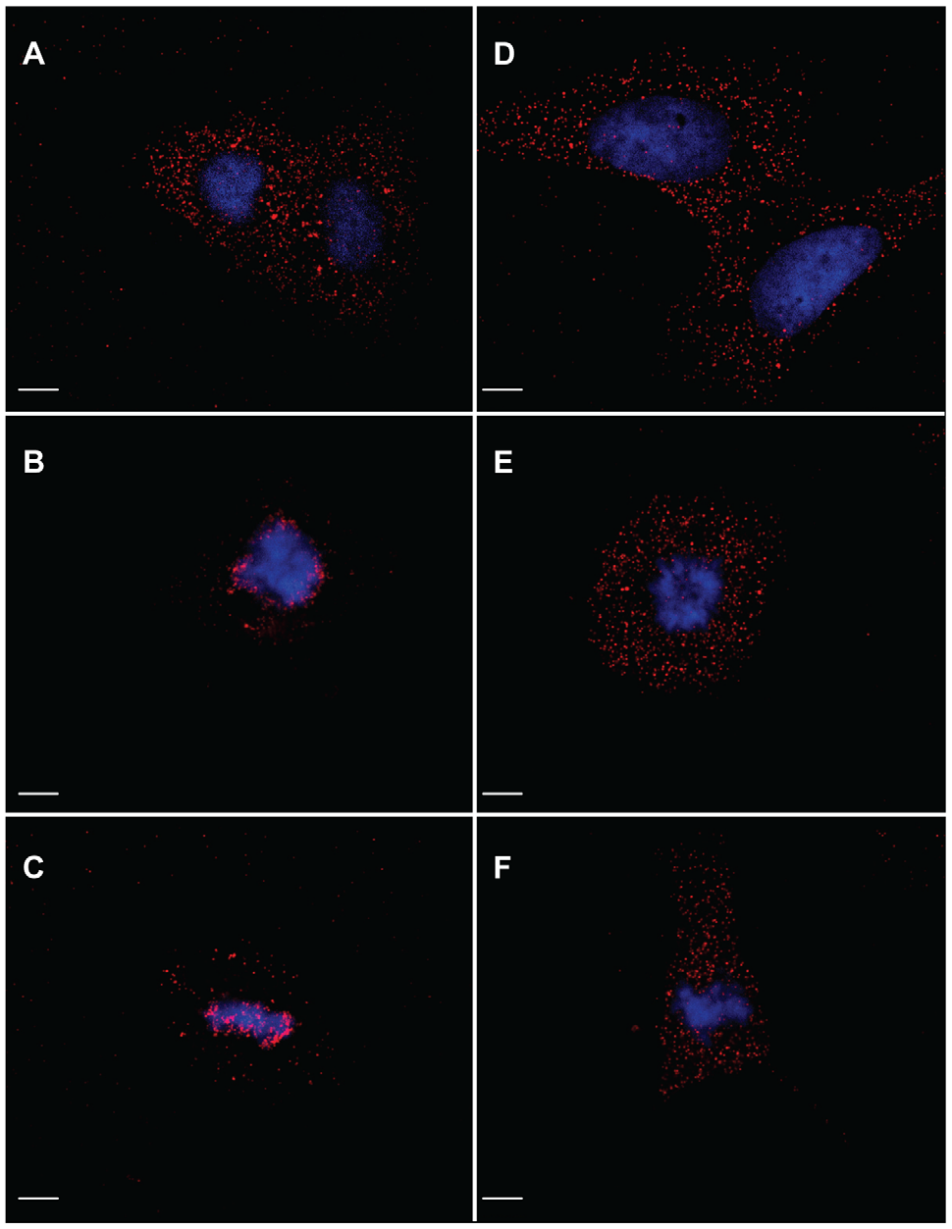
Immunofluorescence analysis of p12 proteins of the 1xMycR and the 1xMycR/PM14 viruses in infected cells. Dividing, non-synchronized U/R cells were infected with 1xMycR (A–C) or with the 1xMycR/PM14 (D–E) viruses. 12 h postinfection the cells were stained and visualized as described in Fig. 4. Interphasic (A and D) and mitotic (B, C, E and F) cells were identified according to the condensation of the chromosomes. Bars represent 10 µm.

Our data provide strong evidence that p12 is a functional component of the PIC. Yet, the exact function of p12 is currently unknown. In principle, p12 may influence one or more steps that include: migration of the PIC along the cytoplasm, nuclear entry and targeting the chromatin for integration. Elaborate biochemical analysis of cells infected with wt MLV or with an integration-defective p12 mutant - the PM14 virus - demonstrated that the two viruses have the same distribution of the viral genomic DNA in cytoplasmic and nuclear fractions; yet no circular genomic DNA was detected for the mutant virus, suggesting that PM14 virus is defective in nuclear steps required for productive infection [3]. However, as the authors suggested, association of the PIC of the PM14 virus to the external side of the nuclear envelope and/or nuclear retention could not be dismissed. We tested if the immunofluorescence procedure that we developed to investigate 1xMycR infection could be applied to the analysis of mutant p12 proteins. For this we introduced the PM14 mutation (Fig. 1) into p12 of the 1xMycR clone to generate the 1xMycR/PM14 virus. We then separately transfected the 1xMycR and the 1xMycR/PM14 clones into 293T cells, harvested the virion-containing supernatants and infected sub-confluent U/R cultures with equal amounts of virions (normalized by an exogenous RT assay). The infected cells were processed for immunofluorescence analysis 12 h postinfection with anti-Myc antibodies to detect the p12 proteins. This analysis revealed that both viruses showed similar intracellular distribution in interphasic cells, where the majority of the p12 proteins were cytoplasmic (Fig. 9). However, a remarkable difference between the wt and the mutant virus was observed in mitotic cells, where the p12 proteins of the 1xMycR/PM14 virus were localized mostly to the cytoplasm, in contrast to the substantial association of the p12 proteins of the 1xMycR virus with the chromosomes (Fig. 9). Further quantification of this phenomenon revealed that in interpahsic cells about 8% of the p12 proteins that were derived from the 1xMycR/PM14 virus overlapped the chromosomes, similar to the value (6%) obtained for the 1xMycR virus (Fig. S8). In mitotic cells, however, where almost 70% of the p12 proteins of the 1xMycR virus overlapped the chromosomes, the p12 proteins with the PM14 mutation showed only 11% overlap - a level that was similar to the one obtained in interphasic cells (Fig. S8). These results demonstrated that the PM14 mutation hindered the movement of the p12 proteins from the cytoplasm towards the chromosomes and strongly suggest a role for p12 in PIC trafficking.

## Discussion

In this work we provide microscopic, genetic and biochemical evidence that the p12 protein - a Gag cleavage product - is a functional constituent of the MLV PIC.

We used fluorescent microscopy analysis to detect Myc-tagged p12 proteins of a replication-competent virus (1xMycR), in infected cells. The comparison between the epitope-tagged virus and the untagged wt virus, and between infected cells that express, or do not express, the mCAT-1 receptor for MLV, allowed us to distinguish unambiguously between background signals and immunofluorescent staining that was related to authentic infection of the incoming virus. This is important since MLV particles with the ecotropic envelope may bind human cells independently of specific receptor-envelope interactions, and such binding can be detected by immunofluorescence [42].

When susceptible U/R and NIH3T3 cells were infected with the 1xMycR virus, our immunofluorescence analysis revealed punctate p12 staining at an early time of infection. This staining was attributed to the incoming virus, and not to newly synthesized Gag proteins since it could be detected as early as two hours postinfection, and in aphidicolin-treated cells, where integration and the subsequent Gag expression are inhibited. Of note, throughout the immunofluorescence experiments, the cells were infected with MOI of approximately 3, yet many of the infected cells exhibited hundreds to thousands of p12-positive dots This likely reflects the fact that for retroviruses only a minute fraction of the virions is infectious [41], [42]. The number of these dots was in a direct correlation with the amount of the virus used for infection. Thus, it is likely that the majority of these dots represent p12 proteins that originated from physical particles that are defective in their infectivity.

The punctate, rather than diffuse, pattern of the staining of p12 suggested that p12 proteins are found in a complex in early stages of the infection cycle. Furthermore, this staining was mainly cytoplasmic in aphidicolin-treated cells or in mitotic cells early after infection, in contrast to its localization adjacent to mitotic chromosomes 12 h postinfection. Thus, the pattern and the distribution of p12 proteins in dividing, and in cell-cycle arrested cells, resemble the one of the MLV genomic DNA [21], suggesting that p12 is part of the MLV PIC. In support of this idea are the findings that whereas p12 staining overlapped the staining of MA or CA in virions, in the infected cells p12 staining co-localized only with that of CA, which unlike the MA protein is thought to be part of the MLV PIC. However, in principle, p12 proteins can migrate towards the cellular chromatin as PIC-independent complexes. Therefore, to better evaluate p12-PIC interaction we combined immunofluorescence with FISH analyses, and detected co-localization of p12 proteins with the viral genomic DNA, both in the cytoplasm and in close proximity to the cellular chromatin of infected cells. Since the viral genomic DNA is the hallmark of the PIC, this co-localization suggested that p12 is indeed part of the PIC. The early detection of punctate p12 staining at two hours postinfection, when almost no FISH signal for the genomic DNA could be detected, hints for organization of p12 proteins in a complex that precede the PIC, most likely in the RTC. Moreover, the appearance of p12 puncta in cells that were infected by MLV VLPs with no encapsidated genomic RNA suggests that the presence of the genomic RNA is not necessary for the early organization of p12 in complexes and their trafficking towards the chromosomes. In this scenario, the protein components of the RTC and the PIC may have intrinsic assembly ability which is independent of the presence of the genomic RNA and/or cDNA.

Our analysis revealed, however, that not all the signals of the p12 proteins and the viral DNA genome overlap, raising the possibility that p12 proteins may function in early stages of infection not as constituents of the PIC but as elements that are separated from this complex; in any scenario, such variation in the association of p12 with the viral genomic DNA may account in part for the fact that only a fraction of MLV particles are infectious. Since our microscopic examination could not unambiguously distinguish between these two scenarios describing p12-PIC interactions, we performed a genetic ‘rescue’ assay to evaluate if p12 proteins exert their activity in *trans*, in respect to the PIC, or as constituents of this complex. In this assay, VLPs that were defective in early steps of infection - between reverse transcription and integration - due to a mutation in p12 (PM14 mutation; [31]) were used together with wt virus (pNCA_IRES-GFP_) to co-infect NIH3T3 cells. The presence of wt p12 proteins in the infected cells did not improve the infectivity of the mutant VLPs and this was true for all wt-to-mutant ratios tested, including conditions where wt p12 proteins were present in a very large excess over the mutant p12 proteins. These results strongly suggest that p12 proteins function as part of the PIC. The co-infection experiments also revealed that excess of pNCA_IRES-GFP_ particles repressed the infection of the VLPs regardless if the latter harbored wt or mutant p12 proteins, and that the level of this inhibition rose with the increase in the pNCA_IRES-GFP_-to-VLPs ratio. This probably reflects a competition between the pNCA_IRES-GFP_ virus and the VLPs on cellular factor(s) needed to establish infection and that are found in limiting amounts. The ecotropic receptor may serve as such a factor since more Neo^r^ colonies were obtained when the pNCA_IRES-GFP_ virus and the VLPs (transducing the Neo^r^ vector) had different (the ecotropic and the VSV-G glycoprotein, respectively), instead of the same, envelopes, resulting in the use of different receptors to initiate infection. However, even in such settings, excess of pNCA_IRES-GFP_ over the VLPs resulted in a dose-dependent reduction in the infectivity of the latter, suggesting that saturable cellular factors other than the ecotropic receptor exist. Notably, the PM14 VLPs were more sensitive to the pNCA_IRES-GFP_-mediated suppression, compared to wt VLPs. This phenomenon may indicate that PM14 VLPs are kinetically slower than wt VLPs in transducing the vector. In this scenario, wt PICs may traffic towards integration faster than the PICs with mutant p12 proteins and accordingly, saturate cellular factors needed for infection, before such factors will be available for the mutant PICs.

The above results suggest that p12 is a constituent of the PIC and thus, it is anticipated that the viral genomic DNA should be co-immunoprecipitated with p12 proteins. Indeed, preferential IP of the genome of the 1xMycR virus with anti-Myc antibodies from infected cells was identified, compared to the controls (made of the wt virus or anti-Flag antibodies), providing strong evidence that p12 proteins are found in a complex with the viral genomic DNA. Further biochemical support to the interaction of p12 with the PIC came from the detection of CA, another component of the MLV PIC [14], in the p12 immunoprecipitates, as CA proteins were immunoprecipitated from lysates of cells that were infected with the 1xMycR virus, using anti-Myc antibodies. It should be noted that we failed to directly detect the Myc-tagged p12 proteins in the pellets where CA proteins were found. This may be the result of the use of a monoclonal antibody (anti-Myc) to detect p12, unlike CA detection that was performed using polyclonal sera that may enhance the sensitivity of the detection. Support for this explanation comes from the fact that Western blot analysis of virions showed an intense signal for CA but a relatively weak signal for p12, although the two proteins are present in equimolar quantities inside the viral particles; the difference in detection sensitivity by Western blot analysis was also observed when only CA, but not p12, was detected in extracts of the 1xMycR-infected cells prior to the IP step (data not shown). Importantly, although this analysis was not sensitive enough to detect the tagged p12 proteins, CA IP was specific since it was observed only when the 1xMycR virus and the anti-Myc antibody were used, and not when the co-IP was performed with controls that included an isotype-matched control antibody (IgG1, anti-Flag) or the untagged, wt virus. In the co-IP experiments, only low amounts of CA were detected in the pellets, compared to its level in the cell lysates. This may reflect a labile p12-CA interaction and/or the possibility that not all of the CA molecules interact with p12 proteins. At this stage, it is not clear whether the p12 and CA proteins are complexed together through direct or indirect interactions; yet, our success to detect this interaction in infected cells but not in extracellular virions implies that at least some of the p12 and CA molecules in the virion form new mutual interactions during or after the uncoating step. In any case, these co-IP experiments provide a biochemical support to the notion that p12 and CA proteins have a cooperative effect in early stages of infection, as was concluded from the analysis of swap mutants between the different domains of the Gag of MLV and its closely related virus - the SNV [37]. Because CA is a component of the MLV PIC and since our analysis suggests that p12 is also a functional constituents of the same complex, the two proteins may act in concert to direct the PIC towards nuclear entry and integration. In line with this idea is the observation, made by Yuan et al. [3], and Lee et al. [37], regarding the phenotypic similarity that exists between wt MLVs that are restricted by the Fv1 restriction factor and mutant MLVs harboring either specific mutations in p12, or p12/CA chimeras derived from MLV and SNV. In each of these cases virus infection is characterized by normal levels of reverse transcription but defective production of genomic circular forms and integration.

If p12 proteins act in directing the PIC towards integration, their function(s) may be required during PIC migration through the cytoplasm, nuclear entry and/or nuclear trafficking. Remarkably, we demonstrated here a clear accumulation of the p12 proteins adjacent to mitotic chromosomes, and this accumulation appeared to increase along the steps of mitosis; ranging from 6% of p12 proteins that overlapped the chromatin during interphase, to about 70% overlap during mitosis. Moreover, the PM14 mutation in p12 that renders the virus integration-defective *in vivo* [3], also hindered the accumulation of p12 proteins with the mitotic chromosomes, as the majority (∼90%) of the puncta of the p12 mutant proteins remained cytoplasmic in dividing cells. These results are in agreement with the postulation, discussed above, that wt PICs are kinetically faster in their trafficking towards integration, than PICs that are derived from the infection of the PM14 virus. Yet, it should be noted that Yuan et al. [3], observed that the distribution of the viral genomic DNA between the cytoplasm and the nuclear fractions was similar for both the wt and the PM14 mutant viruses. While our microscopic analysis revealed such similarity for the p12 content in the nucleus of interphasic cells, our results also showed differential nuclear-cytoplasmic distribution of p12 in mitotic cells, for the two viruses. This difference can be explained by the different methodologies used: whereas our microscopy analysis monitored the infection in individual mitotic cells, in which the nuclear envelope is broken, the biochemical fractionation used by Yuan et al., relayed on the lysis of unsynchronized infected cultures and the subsequent separation of intact nuclei from the cytoplasm. In such conditions, mitotic cells with no intact nuclei can be overlooked, especially if these cells present only a fraction of the unsynchronized culture.

Altogether, our results suggest that p12 proteins are associated with the viral genomic DNA and indicate for a role(s) of p12 along the course of trafficking of this DNA from the cytoplasm to the chromosomes. One possibility of such a role is that p12 interacts with cytoplasmic factors that are required for trafficking towards the nucleus. Such specific factors have not been identified for MLV, but may involve cytoskeleton proteins, as was described for HIV and other retroviruses [3]. In addition, the extensive accumulation of p12 in close proximity to the chromosomes hints for a role of p12 very close to the integration step and may even indicate for a direct function in the integration process itself. However, Yuan et al. [3], have demonstrated that PICs derived from a MLV with a mutation in p12 that render the virus integration-defective *in vivo*, were integration-competent in an *in vitro* assay; demonstrating that a defect in p12 function does not necessarily hamper PIC-mediated integration. Thus, p12 may be needed at the vicinity of the chromatin for steps that precede the integration reaction itself. What could be such a role? For HIV-1, it has been demonstrated that the cellular protein LEDGF is a component of the PIC [10], [11] and may serve to tether the IN proteins to the chromatin [10], [44], [45], [46], [47]. LEDGF interacts with IN proteins derived from several lentiviruses but not with MLV IN [10], [48], and no factor with such tethering activity has been described for MLV. In light of the similarity of the N-termini of p12 and the histone H5 protein [49], and because our microscopic data showed an intimate association of p12 with the chromatin it is tempting to assume that p12 also functions in tethering the MLV PIC to the chromatin. Such a scenario may explain why specific mutations in p12 block MLV integration in live cells but do not interfere with integration into naked DNA, *in vitro* [3].

The ability to detect MLV PICs by immunofluorescence through the detection of p12 proteins provides a new tool that may assist the analysis of several issues concerning MLV infection: (i) such microscopic analysis should complement the biochemical approach used to study p12 mutants and resolve, for example, the question whether PICs of specific p12 mutants are capable of entering the nucleus or are trapped on the external side of the nuclear envelope [3]; (ii) p12 tagging may also be applied for the analysis of the recently discovered human retrovirus, the XMRV, which expresses p12 protein with a high degree of similarity to the MLV p12; suggesting a way to monitor XMRV infection in human cells; (iii) Means were developed to visualize HIV-1 RTC/PIC, which were also exploited to study the interaction of these complexes with the TRIM5α restriction factor [50]. Similarly, tagging p12 proteins of N or B -tropic MLVs with the Myc epitope, as was described here for the 1xMycR virus, should allow the analysis of the restriction of these MLVs by Fv1 [51], and TRIM5α-mediated restriction of N-tropic MLV [52], [53].

In summary, the combination of microscopic, genetic and biochemical assays described here provide strong evidence that p12 is a component of the MLV PIC and this interaction is crucial for the progression of the PIC towards integration.

## Materials and Methods

### Viruses

The pNCS plasmid contains an infectious molecular clone of the Moloney MLV [54], and a simian virus 40 origin of replication in the plasmid backbone. Overlapping PCR was used to insert three tandem repeats of the Myc epitope (EQKLISEEDL), between amino acids 45 and 46 of p12 in pNCS, generating the 3xMyc clone. Virions of a faster replicating revertant, which was derived from the 3xMyc virus, were collected and the genomic RNA was extracted and reverse transcribed with MLV RT and random primers (Promega). The cDNA was amplified by PCR using Ex-Taq (Takara Bio Inc.) and a forward primer (5′CCCAGGTTAAGATCAAGG3′, derived from the matrix sequence), together with a reverse primer (5′CTTGGCCAAATTGGTGGG3′, derived from the capsid sequence). The resulting 875 bp fragment was cloned into pTZ57R (Fermentas) and sequenced, revealing that the revertant virus retained only a single, in-frame copy of the Myc epitope in p12. The cloned PCR fragment was digested with BsrGI and XhoI and the resulting 640 bp fragment, containing the entire p12 and the Myc epitope sequence, was used to replace the BsrGI-XhoI wt sequence in pNCS, to generate the 1xMycR clone. Myc-tagged proteins were detected by Western blot analysis using mouse monoclonal anti-Myc antibody (supernatant of 9E10 hybridoma), and a secondary donkey anti-goat horseradish peroxidase (HRP)-conjugated antibody (Jackson Immunoresearch Laboratories, product no. 705-035-147). The PM5 or PM14 mutations in p12 [31] were introduced by overlapping PCR into the BsrGI-XhoI fragment of pNCS to generate PM5 or PM14 clones, respectively. Overlapping PCR was also used to combine the Myc epitope of the 1xMycR virus with the PM14 mutation (Fig. 1) in the above BsrGI-XhoI fragment to generate the 1xMycR/PM14 clone. pNCA_IRES-GFP_ encodes for a replication-competent MLV, which expresses the green fluorescent protein (GFP) under the translational control of the encephalomyocarditis virus internal ribosome entry site (IRES) [43]. This clone was generously provided by Jeremy Luban (University of Geneva).

To generate MLV VLPs with Myc-tagged p12 proteins, containing or lacking the genomic RNA, the following plasmids and procedure were used: the helper plasmid pVSV.G expresses the VSV-G glycoprotein. The pGag-PolGpt.p12 1xMycR, is a derivative of the pGag-PolGpt helper plasmid that expresses the MLV Gag and Pol proteins, and its p12 sequences was modified to include the Myc epitope tag as in 1xMycR virus (Fig. 1). The pQCXIP plasmid encodes an MLV-based vector (Clontech). The pQCXIPΔ5′ encodes for a defective vector that was generated by deleting an internal BsrGI fragment from pQCXIP, resulting in the removal of the 5′ LTR and the packaging signal. VLPs were generated by transfecting subconfluent 293T cells in 60 mm plates with 10 µg of pQCXIP or pQCXIPΔ5′ together with 7.5 µg of pGag-PolGpt.p12 1xMycR and 2.5 µg of pVSV.G DNAs, using the calcium phosphate procedure. Culture supernatants were harvested two days posttransfection and used for infection.

### Cell cultures

Human embryonic kidney 293T cells, human osteosarcoma U2OS cells, and the mouse NIH3T3 fibroblasts were cultured in Dulbecco's Modified Eagle Medium (DMEM), supplemented with 10% heat-inactivated fetal calf serum (FCS), 2 mM L-glutamine, penicillin (20 U/ml), streptomycin (20 µg/ml) and nystatin (2.5 U/ml), in a humidified incubator at 37v and 5% CO_2_. All tissue culture products were purchased from Biological Industries (Beit Haemek, Israel). To generate human cells stably expressing the murine receptor for MLV, U2OS cells were transfected with 7.5 µg of pCDNA 3.1 Zeo(+) that encode for the murine receptor for MLV (mCAT-1; [39]), using the calcium phosphate precipitation method. Transfected cells were selected in the presence of 100 µg/ml zeocin for two weeks and zeocin-resistant colonies were expanded and screened for their susceptibility to MLV infection. One of the clones (clone #12, named hereafter U/R), was chosen for further experiments since it could efficiently be infected with MLV particles encapsidating a MLV vector that expresses the GFP reporter gene (pQCXIP-gfp-C1 vector [55]; data not shown).

To determine the kinetics of spreading of various MLV clones in NIH3T3 cultures, the cells were either infected or transfected with these clones as indicated. The transfection was carried out using the DEAE-dextran method, as described before [56].

When required, cell synchronization was achieved by arresting cell division before S phase by serum starvation and aphidicolin treatment, as previously described [21] and briefly explained here. On day 1, cells were plated on 13 mm round cover-slips in a 24-well plate at approximately 5% confluency per well. On day 2, cells were serum-starved by removing the media, washing the dish with serum-free media and then adding DMEM containing 0.25% FCS. On day 4, serum was added to a concentration of 10%. 6 h after the addition of serum, aphidicolin (2 µg/ml) was added. After 6 h, the medium was removed and fresh medium containing the virus, 10% serum, polybrene (hexadimethrine bromide; 8 µg/ml) and aphidicolin (2 µg/ml) was added. After 2 h, the virus-containing medium was removed and replaced with 10% serum-containing medium and aphidicolin (2 µg/ml). After an additional 12 h, cells were fixed and subjected to immunofluorescence analysis.

### Immunofluorescence

Cells were grown on 13 mm round cover-slips in a 24-well plate to ∼1.5×10^5^ cells/well and were infected with the indicated virus with multiplicity of infection (MOI) of approximately 3 [virus preparations were estimated to have ∼5×10^6^ infectious units (IU)/ml, based on comparisons of their RT activity, determined by RT exogenous assay [57], to a standard MLV stock with known IU concentration. This stock was made of pNCA_IRES-GFP_, a replication-competent MLV that expresses the GFP marker [43], allowing the determination of its titer by measuring the number of GFP-positive cells in infected cultures, by fluorescence-activated cell sorting (FACS) analysis]. Cells were incubated with the virus for the indicated time, after which they were washed three times with PBS and fixed with 4% paraformaldehyde for 20 min. After three washes with 50 mM glycine in PBS, the cells were permeabilized with 0.1% Triton in PBS for 2 min and immediately washed three times with PBS. The cells were then incubated with blocking solution [1∶10 normal goat serum in Tris-Buffered Saline (TBS; 50 mM Tris-HCl, 150 mM NaCl pH 7.5)] for 30 min, followed by one hour incubation with a mouse monoclonal anti-Myc antibody (supernatant of 9E10 hybridoma, diluted 1∶6 in TBS), and washed once with TBS and twice with PBS (5 min each wash). The cells were then incubated for one hour with a Cy-3-conjugated goat anti-mouse antibody (Jackson Immunoresearch Laboratories, product no. 115-166-072) diluted 1∶500 in TBS and washed once with TBS and twice with PBS (5 min each wash). The nuclei were stained for 20 min at room temperature with DAPI (1 µg/ml in PBS), followed by two washes with PBS. The cover-slips were glued to glass slides with aqueous mounting media containing anti-fading agent (BIOMEDA). All the above steps were carried out at room temperature.

For co-localization experiments of p12 and MA or CA proteins, the immunofluorescence procedure was performed as described above with the following modifications: the blocking solution was made of 3% bovine serum albumin (BSA) in PBS and the supernatant of the 9E10 hybridoma was diluted 1∶6 in PBS. Goat polyclonal anti-MA antiserum (American National Cancer Institute, product no. 78S-282) or goat polyclonal anti-CA antiserum (American National Cancer Institute, product no. 81S-263), were used at a 1∶1000 dilution in PBS containing 3% BSA. These antisera were raised against the MA or the CA proteins of the Rauscher MLV, but cross-react with the MA and CA proteins of the Moloney MLV, respectively. Secondary antibodies included the FITC-conjugated F(ab')_2_ fragment donkey anti-mouse IgG (H+L) (Jackson Immunoresearch Laboratories, product no. 715-096-150), or Red-X- conjugated F(ab')_2_ fragment donkey anti-goat IgG (H+L) (Jackson Immunoresearch Laboratories, product no. 705-296-147), both antibodies were used at a 1∶200 dilution in PBS containing 3% BSA.

BX50 microscope (Olympus), LSM 510 META confocal microscope (Zeiss) or spinning disk confocal (Yokogawa CSU-22 Confocal Head) microscope (Axiovert 200 M, Carl Zeiss MicroImaging) were used in this study where indicated. Quantification of the overlap between p12 and chromatin signals was achieved through the following procedure: Cells, infected with the 1xMycR virus were processed for immunofluorescence analysis as described above. Then, the entire cell volume was imaged by confocal microscopy and the picture was deconvolved with the Nearest Neighbors deconvolution algorithm of SlideBook. Subsequently, three dimensional acquisitions were projected on a two dimensional plane. After this, the specific signals of the p12-based and DAPI-based staining were identified through intensity based segmentation, the total signal intensity was calculated for each signal and the percentage of overlapping signal was deduced by subtraction of the DAPI region of interest (ROI) from the p12 ROI. Approximately 400 dots of p12 signal per image were analyzed. All the above steps were performed employing the SlideBook software (Intelligent Imaging Innovations).

### Combined immunofluorescence and FISH

Cells were grown on 20×20 mm cover-slips in 6-well plates to 30% confluency and were infected with 1 ml media, containing equal amounts of 1xMycR or wt viruses, normalized by RT activity using the exogenous RT assay [57]. Unless otherwise indicated, all the following steps were performed at room temperature. At the indicated time postinfection, slides were washed with PBS, fixed with 4% paraformaldehyde in PBS for 10 min, permeabilized with 0.1% Triton in PBS for 10 min**,** washed for 10 min in 0.1 M Tris pH 7.4, incubated for 20 min in 20% glycerol in PBS, freeze-thawed three times in liquid nitrogen, washed once with PBS and once with 0.1% Triton in PBS, blocked for 30 min with normal goat serum that was diluted 1∶10 with PBS, incubated for 60 min with mouse anti-Myc monoclonal antibody (supernatant of hybridoma 9E10, diluted 1∶6 in TBS), and washed once with TBS for 5 min and twice for 5 min in PBS. Slides were then incubated for 60 min with a Cy-3-conjugated goat anti-mouse antibody (Jackson Immunoresearch Laboratories, product no. 115-166-072, diluted 1∶500) and washed once with TBS and twice with PBS (each wash for 5 min). The slides were re-fixed in 4% paraformaldehyde in PBS for one min, rinsed with PBS and incubated in 70% ethanol overnight. To detect the viral DNA *in situ*, DNA FISH was performed as described previously [58] with the following modifications: The following day, the cover-slips were dried and glued to glass slides with the cells facing up. The slides were then immersed for 10 min in 0.1M HCl, 10 min in 0.5% Triton X-100 in PBS at 37°C and washed three times in PBS. Slides were dehydrated by a series of ethanol washes (70%, 90% and 100%; 5 min each), and incubated at 37°C for 1 hour, followed by denaturation in 70% (v/v) deionized formamide (F9037, Sigma) in 2xSSC, at 75°C, for 5 min. The slides were then briefly washed with 70% ethanol and dehydrated once again in a series of ice-cold ethanol washes (70%, 90% and 100%, 5 min each), air dried and warmed to 37°C. Each slide was spotted with 10 µl of biotin-labeled probe in hybridization solution (see below), sealed with glass cover-slips and rubber cement and incubated in a moist chamber overnight at 37°C. Following hybridization, the sealing was gently removed and the slides were washed three times with 50% (v/v) formamide in 2xSSC (prewarmed to 42°C, 5 min each wash), followed by three washes with 0.1XSSC (prewarmed to 60°C, 5 min each wash). The slides were then incubated with blocking solution (3% BSA in 4xSSC, 30–60 min at 37°C). All stages following blocking were carried out in the dark. The hybridized biotin-labeled probe was detected with FITC-conjugated avidin (A-2011, Vector Laboratories,1∶400 dilution), which was incubated with the slides for 30 min at 37°C in 1% BSA/4xSSC and 0.1% Tween 20. Slides were then washed three times with 4xSSC and 0.1% Tween 20 (prewarmed to 42°C, 5 min each wash), and covered with antifade (VECTASHIELD, Vector Laboratories) containing DAPI (200 ng/ml) under glass cover-slips.

The biotin-labeled probe was prepared in a nick-translation reaction (100 µl, 2 h at 16°C), containing nick-translation buffer (50 mM Tris-HCl pH 7.8, 5 mM MgCl_2_,50 ng/ml BSA), plasmid DNA template (pNCS, 2 µg), dATP, dGTP, dCTP (50 nM of each nucleotide, Sigma), and biotin-11-dUTP (50 nM, Roche), β-mercaptoethanol (10 mM), DNaseI (30 ng/ml, freshly diluted), Klenow polymerase (20 U, New England Biolabs). A sample (8 µl) of this reaction was separated in a 2% agarose gel to verify the generation of a smear, made of approximately 150–500 bp-long DNA products. The rest of the reaction was kept frozen at −20°C until used. On the day of the hybridization the probe was ethanol precipitated with 10 µg of salmon sperm DNA, resuspended in 50 µl of 100% deionized formamide (Sigma, F9037), thoroughly mixed with 50 µl of 20% dextran sulfate in 2xSSC, denatured (75°C, 5 min), and immediately applied to the denatured slides (10 µl/slide).

### Co-immunoprecipitation

For Co-IP of the viral genomic DNA, supernatants of sub-confluent NIH3T3 cultures, chronically infected with the wt or the 1xMycR virus, were harvested, diluted 1∶1 with growth medium and complemented with polybrene (8 µg/ml final concentration). 2 ml of the virus-containing media were used to infect 5×10^6^ NIH3T3 cells in 10 cm plates for 2 h, after which the media were replaced with fresh growth media, followed by additional 5 h incubation. For each IP, 2 plates were trypsinized and the cells were washed once with PBS. Cell pellets were resuspended in 1 ml of IP lysis buffer [50 mM Tris pH 7.5, 150 mM NaCl, 0.5% NP-40, 10 mM MgCl_2_, 1x protease inhibitor cocktail (Roche, product no. 1183614500)] and incubated for 30 min at 4°C with constant agitation. Lysed samples were centrifuged at 20,800 g for 10 min. 5 µl sample of each of the cleared lysates was diluted 1∶10, and triplicates (5 µl each) were analyzed by qPCR. The remaining lysates were then incubated for 30 minutes at 4°C with 50 µl of magnetic polystyrene Dynabeads Protein G (Invitrogen, product no. 100.03D), pre-conjugated to specific antibodies [the magnetic beads were pre-incubated with 200 µl of supernatant of the anti-Myc hybridoma (9E10), or with 0.2 µl (in 200 µl PBS) of anti-Flag monoclonal antibody (Sigma, F1804), for 10 minutes at room temperature with a constant agitation and washed once with PBS]. The samples were placed on a magnet and the beads-free lysates were discarded. The magnetic beads were washed once with IP washing buffer (50 mM Tris pH 7.5, 150 mM NaCl, 0.1% NP-40, 10 mM MgCl_2_), twice with PBS, and then transferred into a new tube. The beads were resuspended in 50 µl TE pH 7.4 (10 mM Tris-Cl pH 7.4, 1 mM EDTA) and 5 µl of the bead slurry were analyzed by PCR with MLV-specific primers (forward primer 5′CCCAGGTTAAGATCAAGG3′, and reverse primer 5′CTTGGCCAAATTGGTGGG3′). For qPCR, triplicates (5 µl each) of the bead slurry were analyzed; real-time PCR reactions were performed with MLV-specific primers (forward primer 5′-AGCCCTTTGTACACCCTAAGC-3′ and reverse primer 5′-GAGGTTCAAGGGGGAGAGAC-3′) and Fast SYBR Green Master Mix (Applied Biosystems, product no. 4385612), and analyzed with StepOnePlus Real-Time PCR System (Applied Biosystems). Standard curves were used to determine the absolute DNA quantity in the samples. To calculate the relative efficiency of the immunoprecipitation of the viral genomic DNA in the different experimental settings, the levels of the viral genomic DNA in the cell extracts and in the IP pellets were quantified by qPCR, and the background signal obtained in the ‘mock-infected’ sample was subtracted. The level of the immunoprecipitated viral genomic DNA (IP sample) was divided by the level of this DNA in the cell lysate (input sample), to give normalized IP value. The normalized value for the genome of the 1xMycR virus that was immunoprecipitated with anti-Myc antibodies was set as 100% and was compared to the normalized values obtained for the genome of the 1xMycR virus that was immunoprecipitated with anti-Flag antibodies, and for the genome of the wt virus that was immunoprecipitated with anti-Myc antibodies. The average value of this comparison, obtained from three independent Co-IP experiments, gave the ‘Relative IP Efficiency’ index.

For Co-IP of CA, infections and lysis of the infected cells were carried out as described above. To reduce non-specific binding to the agarose beads that were used for this procedure, the cell lysates were first incubated for 30 minutes at 4°C with a mixture of 7.5 µl of protein A-agarose beads (Roche, product no. 11 134 515 001) and 7.5 µl of protein G-agarose beads (Roche, product no. 11 243 233 001), Then, the lysate-bead slurries were centrifuged at 20,800 g for 2 minutes, the beads were discarded and the cleared supernatants were incubated overnight at 4°C with 15 µl protein A and 15 µl G beads, pre-conjugated to specific antibodies [beads were pre-incubated with 0.5 ml of supernatant of the anti-Myc hybridoma (9E10) or with 0.5 µl (in 0.5 ml of PBS) of anti-Flag monoclonal antibody (Sigma, F1804)]. Samples were centrifuged at 10,000 rpm for 2 min at 4°C and the pelleted beads were washed once with IP washing buffer, twice with PBS, and directly boiled in 2x Sample Buffer. The CA protein was detected by Western blot, using goat anti-MLV CA polyclonal antibody (National Cancer Institute, product no. 81S-263) and a secondary donkey anti-goat HRP-conjugated antibody (Jackson Immunoresearch Laboratories, product no. 705-035-147). For Co-IP of CA from extracellular virions, supernatants of sub-confluent NIH3T3 cultures, chronically infected with the wt or the 1xMycR virus, were harvested and virions were purified from 2 ml of undiluted medium by ultracentrifugation (107,000 *g* for 2 h) through 25% sucrose cushions. Lysis of the virion pellets and CA Co-IP were performed as described above for the infected cells.

### Phylogenetic analysis

The p12 domain of the Gag polyprotein of the Moloney MLV (SWISS-PROT: P03332) was aligned with 12 homologous sequences that were retrieved from the SWISS-PROT database, using a PSI-BLAST search. The sequences were then multiple aligned (MSA) using the CLUSTALW program, and a phylogenetic tree consistent with the MSA was constructed. Calculation of the conservation scores for each residue was carried out by the Rate4Site algorithm [59], which is based on the Maximum Likelihood method. All these stages were curried out automatically by the ConSeq server (http://conseq.tau.ac.il/), as described in [60].

## Supporting Information

Figure S1 The number of p12 puncta in infected cells correlates with the amount of virions used for infection. U/R cells were infected with serial dilutions of the 1xMycR virus and were processed for immunofluorescence. For each dilution, the number of p12 puncta in infected cells was visualized using spinning disk confocal (Yokogawa CSU-22 Confocal Head) microscope (Axiovert 200 M, Carl Zeiss MicroImaging), and the number of objects (puncta) in the inspected cells was determined employing the SlideBook software (Intelligent Imaging Innovations). Then, the average number of p12 puncta per cell, the standard deviation and the standard error of the mean were calculated (A). The log of the average number of p12 puncta per cell was plotted against the log of the dilutions (B). The trend line and its R-squared value were calculated and drawn using the Microsoft Excel software.
(0.15 MB TIF)

Figure S2 Quantification of the overlap between the p12 proteins and the chromosomes. U/R cells were infected with 1xMycR and p12 detection, as well as the staging of the cell cycle of the infected cells were performed as described in Fig. 4. The percentages of the p12 signal that overlapped the DAPI signal were calculated using the SlideBook software (Materials and Methods) and are presented as columns with standard error bars. ‘n’ denotes the number of individual cells inspected for each stage of the cell cycle.
(0.12 MB TIF)

Figure S3 Detection of the MLV genome in infected cells by FISH. U/R (A–C) or U20S (D) cells were infected with the wt virus (A, B and D), or were mock-infected with virus-free medium (C). 12 h postinfection the cells were processed for FISH analysis, using MLV-derived, biotin-labeled probe that was detected with a Cy3-conjugated avidin (ExtrAvidin-Cy3 Conjugate; Sigma, E4142). The cells were visualized with a BX50 microscope (Olympus). Bars represent 10 µm.
(2.16 MB TIF)

Figure S4 Detection of viral p12 proteins and genomic DNA by immunofluorescence combined with FISH. U/R cells were infected and processed for immunofluorescence combined with FISH, as described in Fig. 6. The entire cell volume was imaged with a LSM 510 META confocal microscope (Zeiss). Micrograph depicts a middle Z section (the 6th of 11 sections) of the nucleus of a dividing cell. This section is visualized in the *x,y* plane (large square), *x,z* plane (upper rectangle) and *y,z* plane (side rectangle). Arrows point to doubly-labeled puncta (green and red yielding a yellow signal) that localize to the center of the condensed chromatin (blue). The image was created with the LSM Image Browser software (Zeiss).
(2.99 MB TIF)

Figure S5 Quantification of the fluorescent puncta in immunofluorescence combined with FISH analysis. U/R cells were infected with 1xMycR virus and processed at the indicated time postinfection for immunofluorescence combined with FISH analysis, as described in Fig. 6. Microscopic images of the cells were inspected manually for fluorescent puncta, to determine the p12 (red), viral DNA (green) and the overlap (yellow) fluorescence of each dot (upper table). Numbers in brackets represent the number of fluorescent dots that overlapped the DAPI staining of the chromosomes. The numbers of the dots with the red, green or yellow fluorescence are presented as percentages of the total number of the fluorescent dots, at each time point postinfection, (columns chart). Quantification of the fluorescence of the extracellular puncta is presented at the lower table.
(0.23 MB TIF)

Figure S6 Generation of p12 puncta in infected cells is independent of the presence of the genomic RNA in infecting particles. 293T cells were transfected with a mixture of the helper plasmids pGag-PolGpt.p12 1xMycR and pVSV.G; this mixture also included either the pQCXIP plasmid encoding an MLV-based vector, or its derivative - the pQCXIPΔ5′ plasmid that contains a defective vector, lacking both the 5′ LTR and the packaging signal. This defective vector was used to generate VLPs with no packaged genomic RNA. Two days posttransfection, equal amounts of virions in the culture supernatants (normalized by an exogenous RT assay) were used to infect U/R cells. p12 was visualized by immunofluorescence as described in Fig. 3. Shown are representative images of interphasic (A, B, D and E) and mitotic (C and E) cells, infected with VLPs containing (A-C) or lacking (D–F) the genomic RNA. Bars represent 10 µm.
(3.00 MB TIF)

Figure S7 Co-IP of p12 with the viral genomic DNA and CA proteins. (A) qPCR analysis of the level of the genomic DNA of the indicated viruses in the lysates of infected cells (‘Input’), and in samples of the magnetic beads (‘IP’) that were coupled to the indicated antibodies. Each value represents the average of three triplicates of the tested sample. For Co-IP of CA, supernatants of NIH3T3 cultures, chronically infected with the indicated virus, were harvested and 2 ml were used to infect naïve NIH3T3 cells. Virions from additional 2 ml culture supernatants were purified by ultracentrifugation through 25% sucrose cushions. Lysates were prepared both from the extracellular virions and from the infected cells and were subject to IP as described in Fig. 7C. 5% of the virion or cell lysate samples and 100% of the IP samples were examined by Western blot analysis, to detect CA in lysates and IP pellets of virions (B) and infected cells (C). ‘1xMycR*’ indicates the samples of 1xMycR-infected cells and 1xMycR virions, used for IP reactions with anti-Flag antibodies.
(0.78 MB TIF)

Figure S8 Quantification of the overlap between p12 and the chromosomes in 1xMycR or 1xMycR/PM14 -infected cells. U/R cells were infected with 1xMycR or 1xMycR/PM14 viruses and p12 detection, as well as the staging of the cell cycle of the infected cells were performed as described in Fig. 4. The percentages of the p12 signal that overlapped the DAPI signal were calculated using the SlideBook software as described in Fig. S2 and are presented as columns with standard error bars. For the analysis of p12 puncta derived from the 1xMycR/PM14 virus, 2 and 10 interphasic and mitotic cells were inspected, respectively; the results of this analysis were compared to the ones obtained for the 1xMycR virus in Fig. S2. For both infections, ‘mitosis’ refers to the average overlap that was calculated for dividing cells regardless of their specific stage in mitosis. Student's t-test analyses show highly significant differences in the overlap of p12 with chromosomes between mitotic and interphasic cells for the 1xMycR virus (p<10^−15^); similarly highly significant differences in the overlap were observed amongst the 1xMycR or 1xMycR/PM14 viruses in mitotic cells (p<10^−17^); and an absence of such differences for either the two viruses in interphasic cells or for the 1xMycR/PM14 in mitotic and interphasic cells (p>0.4).
(0.10 MB TIF)
