# Supplementary figures and images for: The Gag Cleavage Product, p12, is a Functional Constituent of the Murine Leukemia Virus Pre-Integration Complex
Source: PLoS Pathog. 2010 Nov 11;6(11):e1001183. doi: 10.1371/journal.ppat.1001183 (PMC2978732; doi:10.1371/journal.ppat.1001183)

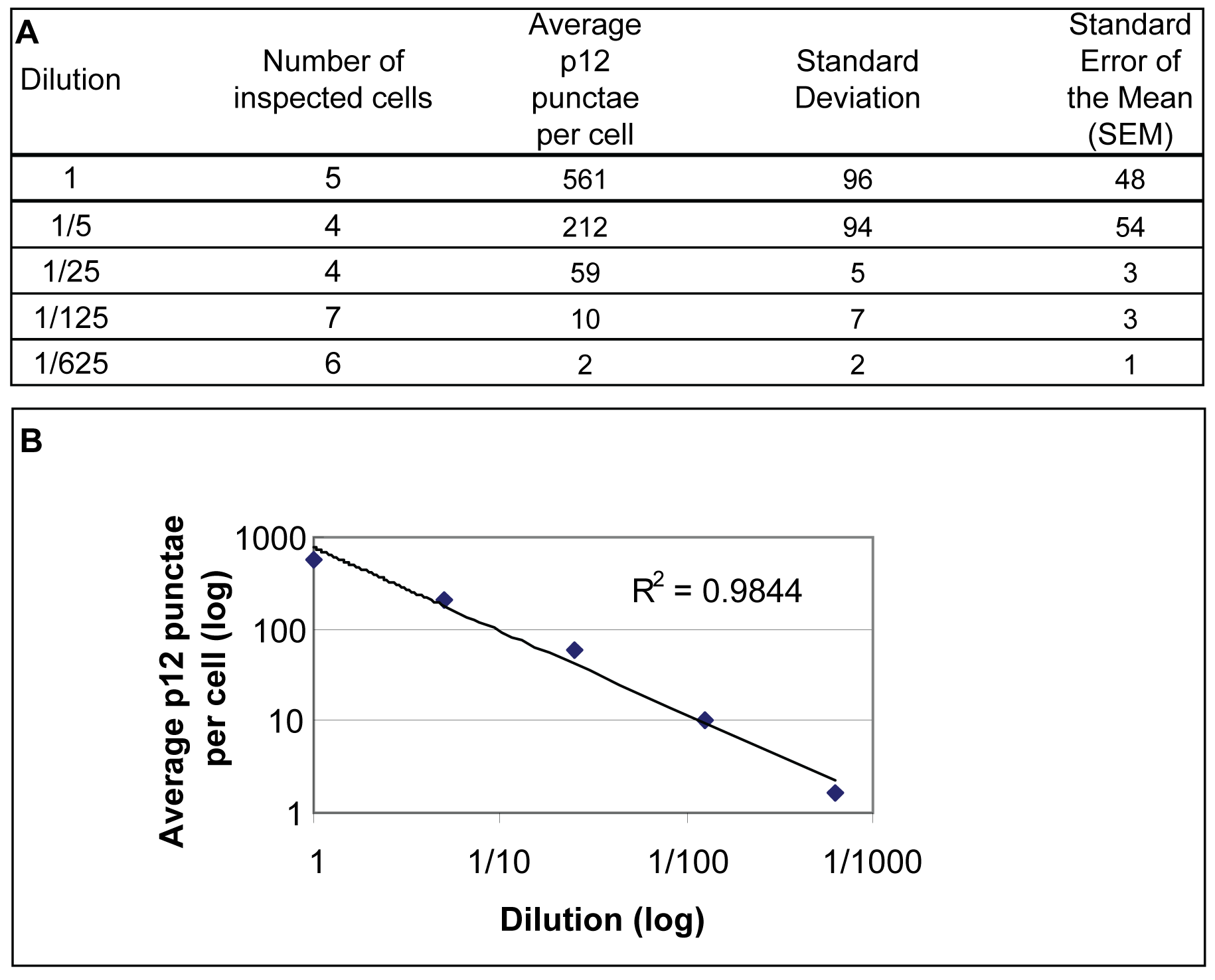

Supplement: Figure S1 — The number of p12 puncta in infected cells correlates with the amount of virions used for infection. U/R cells were infected with serial dilutions of the 1xMycR virus and were processed for immunofluorescence. For each dilution, the number of p12 puncta in infected cells was visualized using spinning disk confocal (Yokogawa CSU-22 Confocal Head) microscope (Axiovert 200 M, Carl Zeiss MicroImaging), and the number of objects (puncta) in the inspected cells was determined employing the SlideBook software (Intelligent Imaging Innovations). Then, the average number of p12 puncta per cell, the standard deviation and the standard error of the mean were calculated (A). The log of the average number of p12 puncta per cell was plotted against the log of the dilutions (B). The trend line and its R-squared value were calculated and drawn using the Microsoft Excel software. (0.15 MB TIF) [file ppat.1001183.s001.tif]

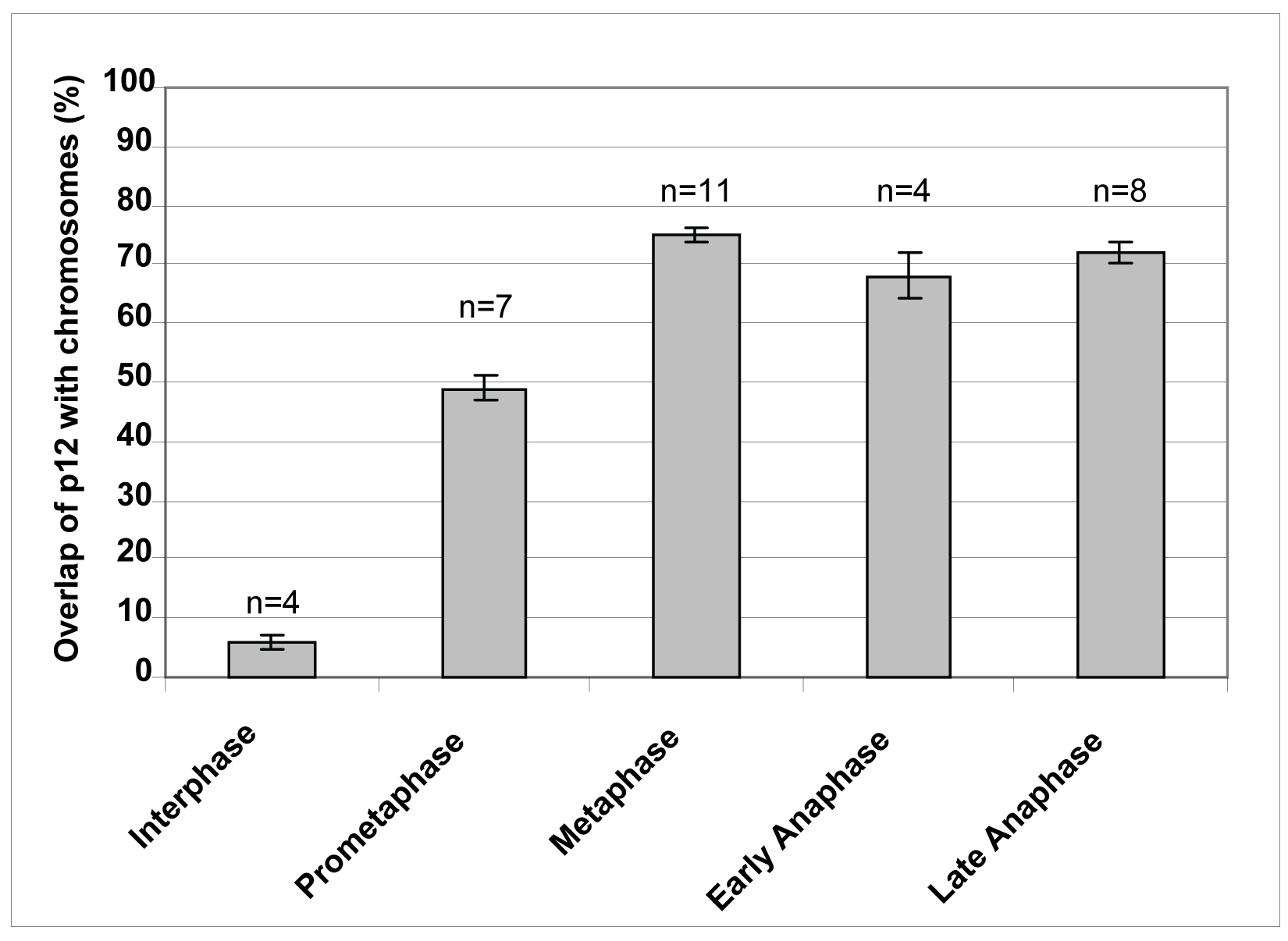

Supplement: Figure S2 — Quantification of the overlap between the p12 proteins and the chromosomes. U/R cells were infected with 1xMycR and p12 detection, as well as the staging of the cell cycle of the infected cells were performed as described in Fig. 4. The percentages of the p12 signal that overlapped the DAPI signal were calculated using the SlideBook software (Materials and Methods) and are presented as columns with standard error bars. ‘n’ denotes the number of individual cells inspected for each stage of the cell cycle. (0.12 MB TIF) [file ppat.1001183.s002.tif]

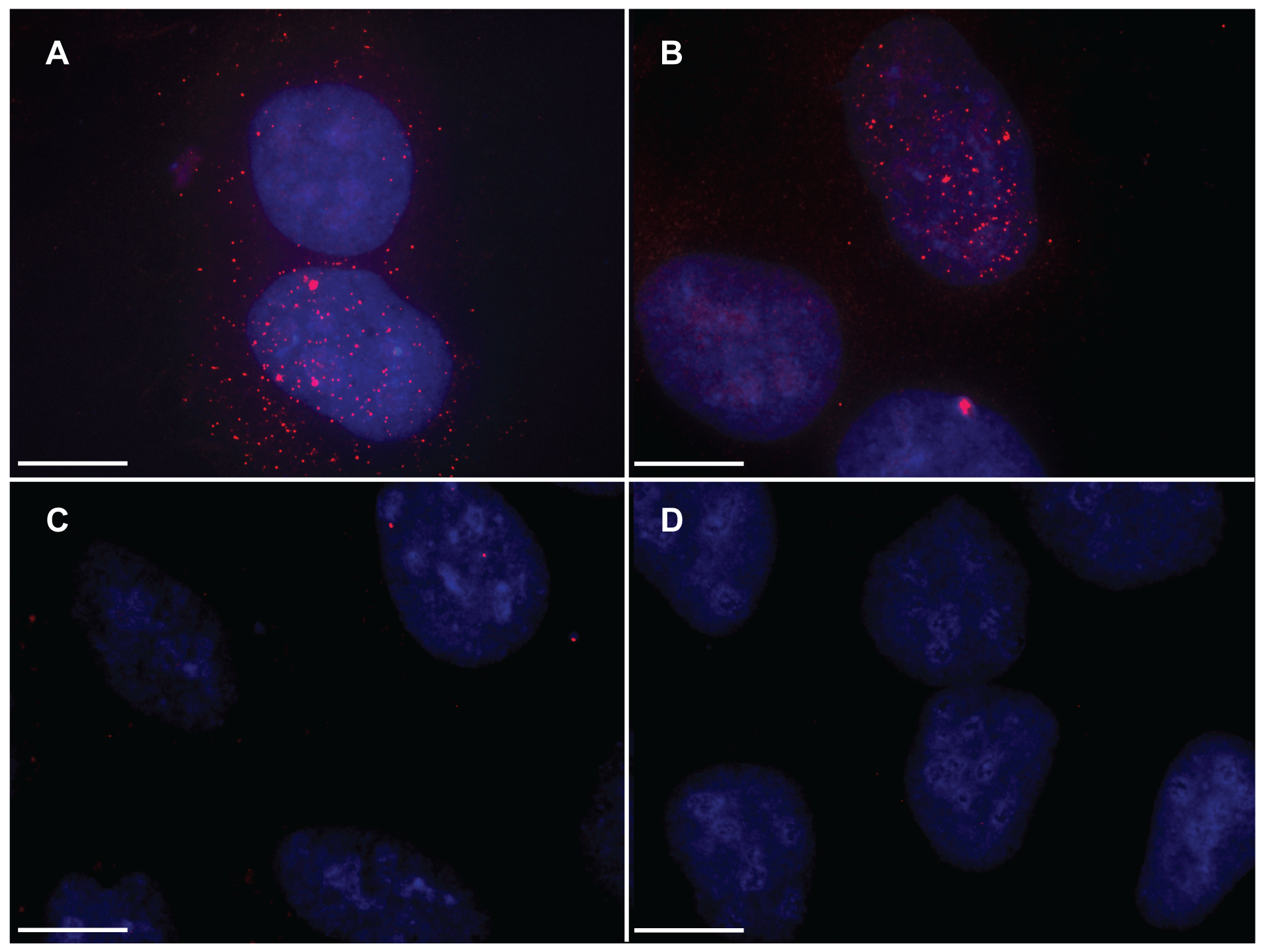

Supplement: Figure S3 — Detection of the MLV genome in infected cells by FISH. U/R (A–C) or U20S (D) cells were infected with the wt virus (A, B and D), or were mock-infected with virus-free medium (C). 12 h postinfection the cells were processed for FISH analysis, using MLV-derived, biotin-labeled probe that was detected with a Cy3-conjugated avidin (ExtrAvidin-Cy3 Conjugate; Sigma, E4142). The cells were visualized with a BX50 microscope (Olympus). Bars represent 10 µm. (2.16 MB TIF) [file ppat.1001183.s003.tif]

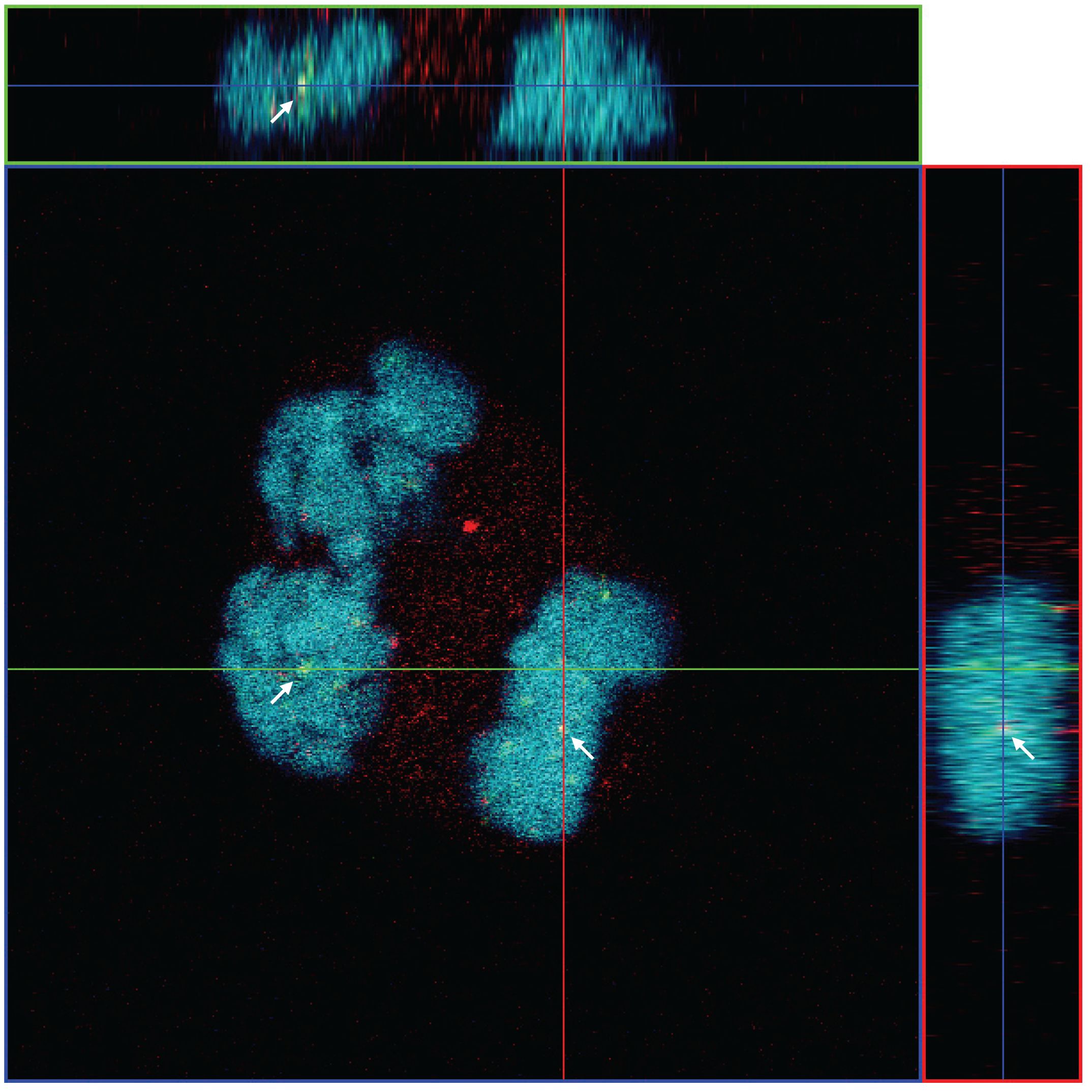

Supplement: Figure S4 — Detection of viral p12 proteins and genomic DNA by immunofluorescence combined with FISH. U/R cells were infected and processed for immunofluorescence combined with FISH, as described in Fig. 6. The entire cell volume was imaged with a LSM 510 META confocal microscope (Zeiss). Micrograph depicts a middle Z section (the 6th of 11 sections) of the nucleus of a dividing cell. This section is visualized in the x,y plane (large square), x,z plane (upper rectangle) and y,z plane (side rectangle). Arrows point to doubly-labeled puncta (green and red yielding a yellow signal) that localize to the center of the condensed chromatin (blue). The image was created with the LSM Image Browser software (Zeiss). (2.99 MB TIF) [file ppat.1001183.s004.tif]

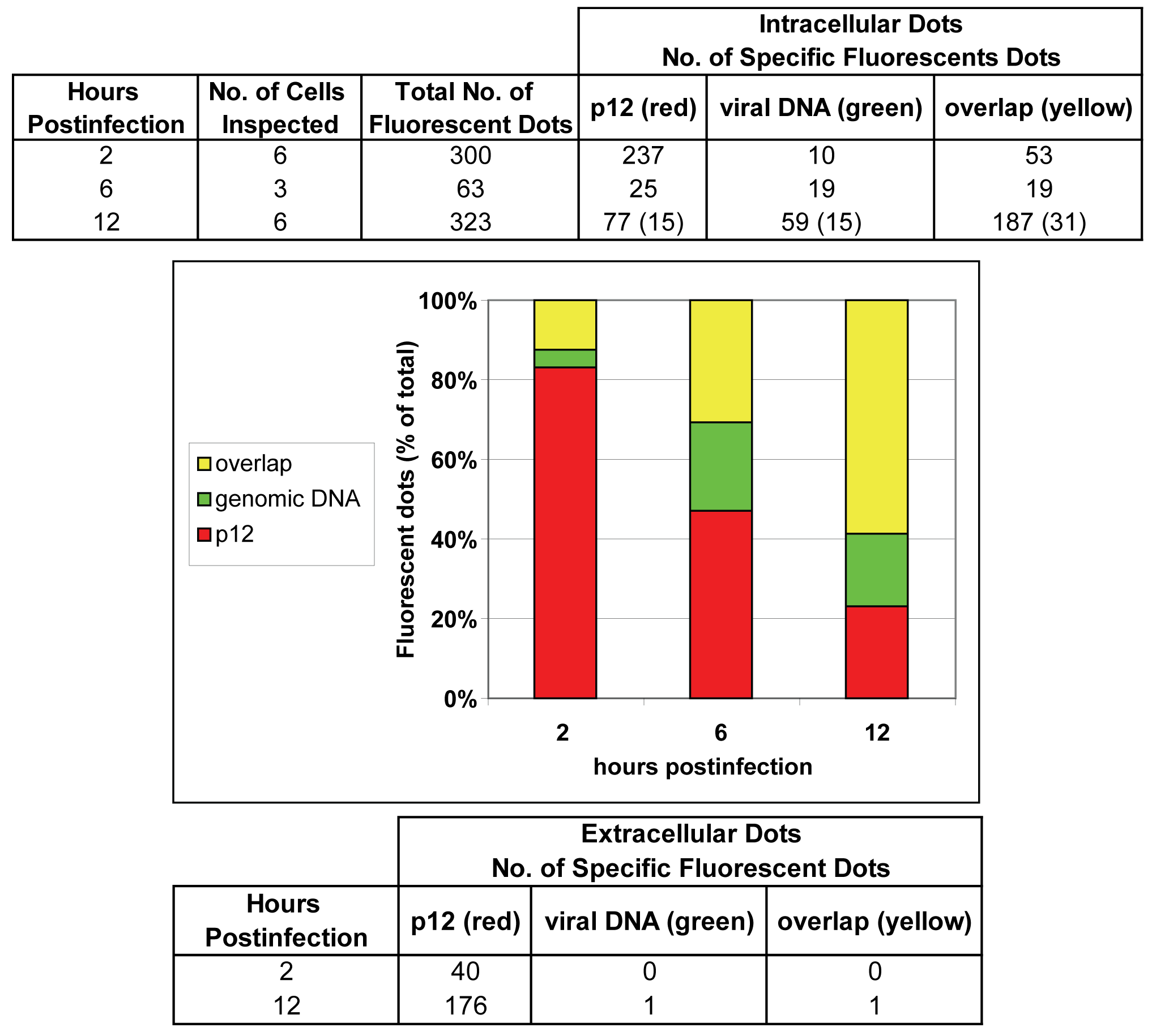

Supplement: Figure S5 — Quantification of the fluorescent puncta in immunofluorescence combined with FISH analysis. U/R cells were infected with 1xMycR virus and processed at the indicated time postinfection for immunofluorescence combined with FISH analysis, as described in Fig. 6. Microscopic images of the cells were inspected manually for fluorescent puncta, to determine the p12 (red), viral DNA (green) and the overlap (yellow) fluorescence of each dot (upper table). Numbers in brackets represent the number of fluorescent dots that overlapped the DAPI staining of the chromosomes. The numbers of the dots with the red, green or yellow fluorescence are presented as percentages of the total number of the fluorescent dots, at each time point postinfection, (columns chart). Quantification of the fluorescence of the extracellular puncta is presented at the lower table. (0.23 MB TIF) [file ppat.1001183.s005.tif]

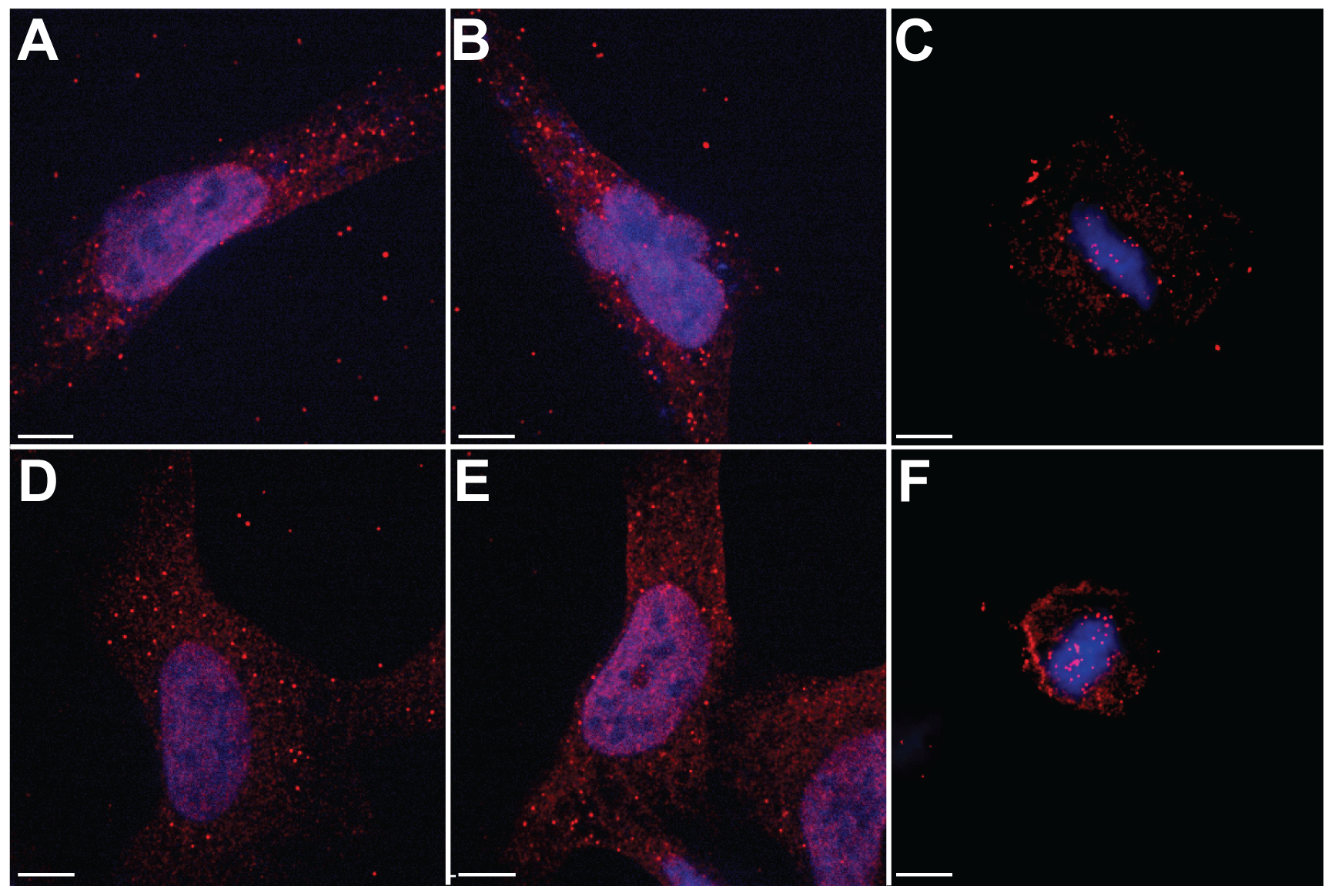

Supplement: Figure S6 — Generation of p12 puncta in infected cells is independent of the presence of the genomic RNA in infecting particles. 293T cells were transfected with a mixture of the helper plasmids pGag-PolGpt.p12 1xMycR and pVSV.G; this mixture also included either the pQCXIP plasmid encoding an MLV-based vector, or its derivative - the pQCXIPΔ5′ plasmid that contains a defective vector, lacking both the 5′ LTR and the packaging signal. This defective vector was used to generate VLPs with no packaged genomic RNA. Two days posttransfection, equal amounts of virions in the culture supernatants (normalized by an exogenous RT assay) were used to infect U/R cells. p12 was visualized by immunofluorescence as described in Fig. 3. Shown are representative images of interphasic (A, B, D and E) and mitotic (C and E) cells, infected with VLPs containing (A-C) or lacking (D–F) the genomic RNA. Bars represent 10 µm. (3.00 MB TIF) [file ppat.1001183.s006.tif]

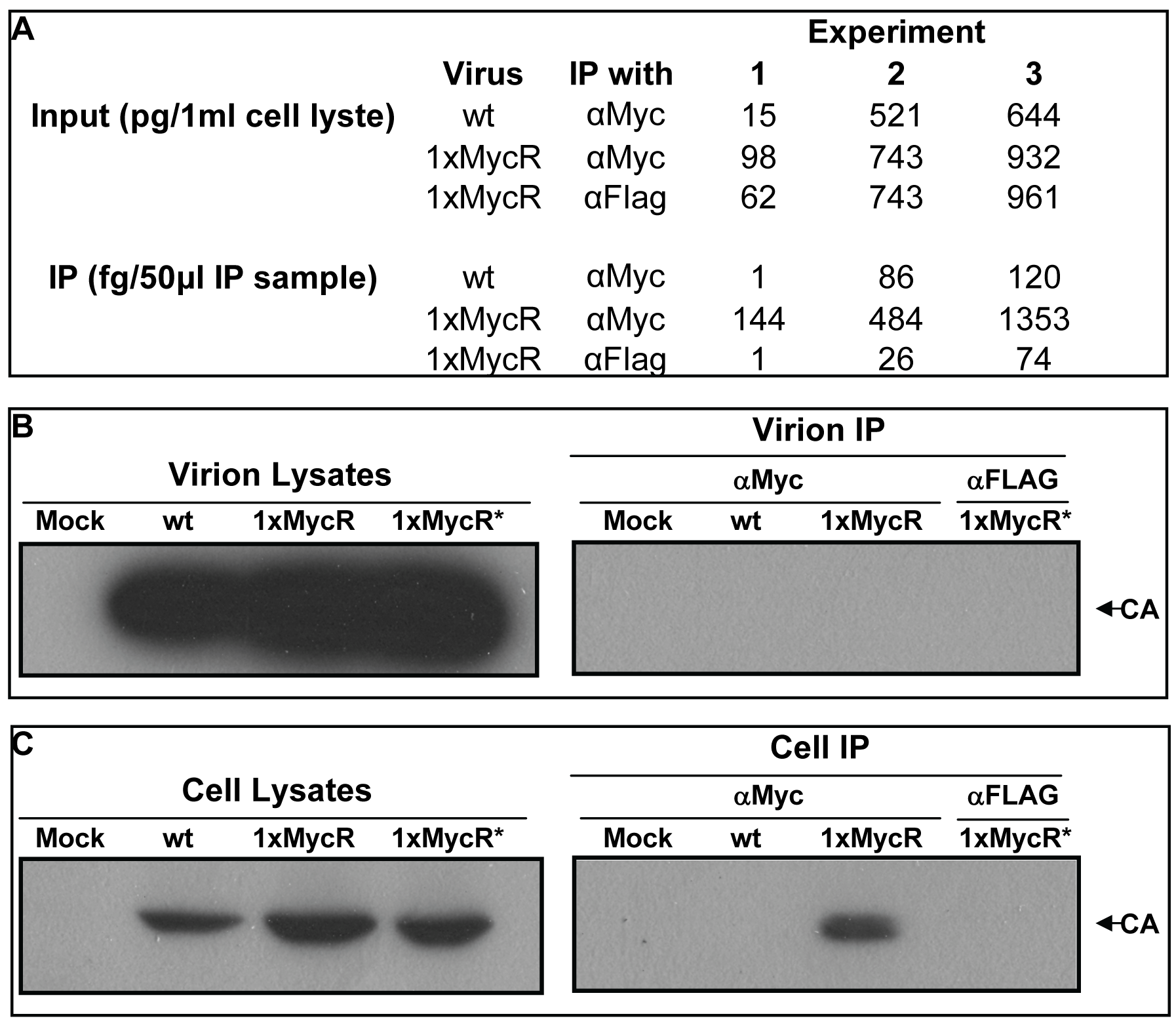

Supplement: Figure S7 — Co-IP of p12 with the viral genomic DNA and CA proteins. (A) qPCR analysis of the level of the genomic DNA of the indicated viruses in the lysates of infected cells (‘Input’), and in samples of the magnetic beads (‘IP’) that were coupled to the indicated antibodies. Each value represents the average of three triplicates of the tested sample. For Co-IP of CA, supernatants of NIH3T3 cultures, chronically infected with the indicated virus, were harvested and 2 ml were used to infect naïve NIH3T3 cells. Virions from additional 2 ml culture supernatants were purified by ultracentrifugation through 25% sucrose cushions. Lysates were prepared both from the extracellular virions and from the infected cells and were subject to IP as described in Fig. 7C. 5% of the virion or cell lysate samples and 100% of the IP samples were examined by Western blot analysis, to detect CA in lysates and IP pellets of virions (B) and infected cells (C). ‘1xMycR*’ indicates the samples of 1xMycR-infected cells and 1xMycR virions, used for IP reactions with anti-Flag antibodies. (0.78 MB TIF) [file ppat.1001183.s007.tif]

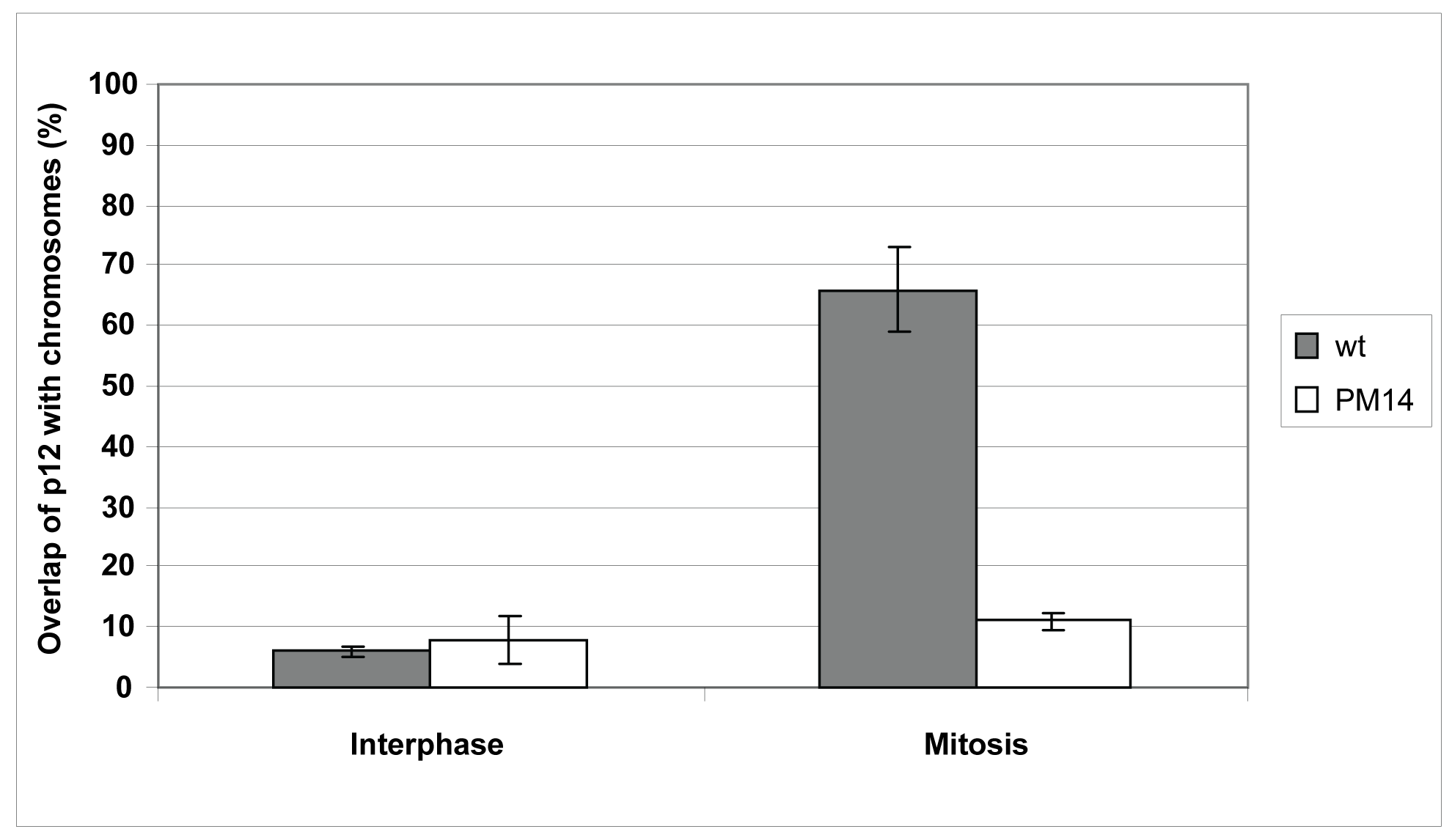

Supplement: Figure S8 — Quantification of the overlap between p12 and the chromosomes in 1xMycR or 1xMycR/PM14 -infected cells. U/R cells were infected with 1xMycR or 1xMycR/PM14 viruses and p12 detection, as well as the staging of the cell cycle of the infected cells were performed as described in Fig. 4. The percentages of the p12 signal that overlapped the DAPI signal were calculated using the SlideBook software as described in Fig. S2 and are presented as columns with standard error bars. For the analysis of p12 puncta derived from the 1xMycR/PM14 virus, 2 and 10 interphasic and mitotic cells were inspected, respectively; the results of this analysis were compared to the ones obtained for the 1xMycR virus in Fig. S2. For both infections, ‘mitosis’ refers to the average overlap that was calculated for dividing cells regardless of their specific stage in mitosis. Student's t-test analyses show highly significant differences in the overlap of p12 with chromosomes between mitotic and interphasic cells for the 1xMycR virus (p<10−15); similarly highly significant differences in the overlap were observed amongst the 1xMycR or 1xMycR/PM14 viruses in mitotic cells (p<10−17); and an absence of such differences for either the two viruses in interphasic cells or for the 1xMycR/PM14 in mitotic and interphasic cells (p>0.4). (0.10 MB TIF) [file ppat.1001183.s008.tif]
